# Emerging Trends in Nanotechnology: Aerogel-Based Materials for Biomedical Applications

**DOI:** 10.3390/nano13061063

**Published:** 2023-03-15

**Authors:** Noremylia Mohd Bakhori, Zarini Ismail, Mohamad Zaki Hassan, Rozzeta Dolah

**Affiliations:** 1 Faculty of Medicine and Health Sciences, Universiti Sains Islam Malaysia, Persiaran Ilmu, Putra Nilai, Nilai 71800, Negeri Sembilan, Malaysia; 2 Razak Faculty of Technology and Informatics, Universiti Teknologi Malaysia, Jalan Sultan Yahya Petra, Kuala Lumpur 54100, Selangor, Malaysia; 3 Department of Chemical Engineering, Universiti Teknologi Malaysia, Jalan Sultan Yahya Petra, Kuala Lumpur 54100, Selangor, Malaysia

**Keywords:** aerogel, silica, biopolymer, biomedical application, wound healing, drug delivery

## Abstract

At present, aerogel is one of the most interesting materials globally. The network of aerogel consists of pores with nanometer widths, which leads to a variety of functional properties and broad applications. Aerogel is categorized as inorganic, organic, carbon, and biopolymers, and can be modified by the addition of advanced materials and nanofillers. Herein, this review critically discusses the basic preparation of aerogel from the sol–gel reaction with derivation and modification of a standard method to produce various aerogels for diverse functionalities. In addition, the biocompatibility of various types of aerogels were elaborated. Then, biomedical applications of aerogel were focused on this review as a drug delivery carrier, wound healing agent, antioxidant, anti-toxicity, bone regenerative, cartilage tissue activities and in dental fields. The clinical status of aerogel in the biomedical sector is shown to be similarly far from adequate. Moreover, due to their remarkable properties, aerogels are found to be preferably used as tissue scaffolds and drug delivery systems. The advanced studies in areas including self-healing, additive manufacturing (AM) technology, toxicity, and fluorescent-based aerogel are crucially important and are further addressed.

## 1. Introduction

Aerogel is a nanostructured material that is gaining popularity as a structural alternative for insulation in a variety of uses, ranging from residences and commercial structures to offshore platforms and spacecraft. Aerogel insulator is thought to provide 40 times the shielding effect of fiber glass, allowing it to be used in space-constrained applications. It is a low-density, high dielectric strength, high specific surface areas, low thermal conductivities, and extremely porous foam with interconnected nanostructures [1,2]. Aerogel is composed of approximately 99.8 percent space, giving it a spectral look, and garnering the name of ‘solid smoke’ [3]. It is typically composed of silica and may take numerous shapes. However, organic polymers, inorganic, carbon allotropes, polysaccharides, transition metals, and nanostructures of semiconductors may also synthesize aerogels [4]. Aerogel is created by drying gels at extremely elevated heat.

In the early 1930s, Kistler and Learned invented the first aerogel by supercritical drying a wet gel and extracting the liquid [5]. It was employed as a tobacco filler and thickener, whereas silica aerogel was used as a thermal insulating blanket. Despite the numerous benefits that silica as well as other inorganic compounds can bring in the production of aerogel, conventional aerogel raw resources are still derived from petrochemical sources. On the other hand, the difficult multistage preparation method stymied the development of aerogel. Nonetheless, native aerogel with a single element is typically afflicted by serious issues such as weak mechanical properties, and a lack of functionalities. The name “aerogel” resurfaced in the 1970s, with the rising use of sol–gel synthesis processes and the usage of aerogel to store rocket fuels [6]. Following that, important efforts were made to simplify the synthesis methods, particularly drying to achieve a low-cost and simple synthesis of aerogel. This paved the way for a wide range of aerogel to be used in various fields of application due to their open structure and lightweight [5,7,8]. To improve aerogel performance, significant growth in the emergence of future aerogel with varied physicochemical features and functional abilities is required [9,10]. For example, aerogel-based biomaterials are now made from a variety of sources or components that imitate the structure of a biological extracellular matrix. The tissues that surround this structure serve as support cells and are affected biochemically by it. Even though an aerogel network has also hybridized with a wide variety of nanostructures and improved functional properties such as antifungal or antimicrobial performance.

Asia-Pacific had the greatest share of the aerogel market in 2018 at 52.2%, and is projected to increase at a compound annual growth rate (CAGR) of 46.3% to reach USD 261.6 million by 2024. Meanwhile, the severe effects of the COVID-19 outbreak may hinder the world economy and reduce the supply of aerogel [11]. Many characteristics of aerogel contribute to their potential interest in biomedical applications, including high surface area, low density, higher absorption rates, and light weight. However, before ever being considered for any medical technology, the biocompatibility of a given aerogel should first be evaluated. Current biomedical challenges are intertwined with the new global social sanitation resulting from dramatic changes and the emergence of a new population lifestyle. Innovative materials and methods must provide a remedy to improve quality of life as the older population and the severity of chronic diseases (cancer, high blood pressure, heart attack) increases. Considering the most recent demographic projection of longevity, high-performance, outstanding, trustworthy, and reproducible goods are aggressively pursued by lengthening their lifetime efficacy and promoting more responsible medication use (antibiotics). However, a knowledge gap in important multidisciplinary scientific fields (materials science, biology, legal considerations, manufacturing technology) may jeopardize the establishment of next-generation pharmaceuticals and tissue transplants capable of satisfying new societal demands [12]. Aerogel-based materials for innovative nanostructured are interesting possibilities for solving these difficulties and surpassing present constraints. Several studies on the production of aerogels have recently been published, with material preparation through prospective applications but with limited discussion on potential applications. Herein, this review outlines the types of aerogels, aerogel synthesis and its respective sources. Then, their reported biocompatibility and biomedical applications in the literatures are also discussed as it covered most of the applications such as drug-delivery carriers, anti-toxicity, antioxidants, bone regenerative, cartilage tissue repair and dentistry.

## 2. Type of Aerogels and Properties

**Figure 1:**
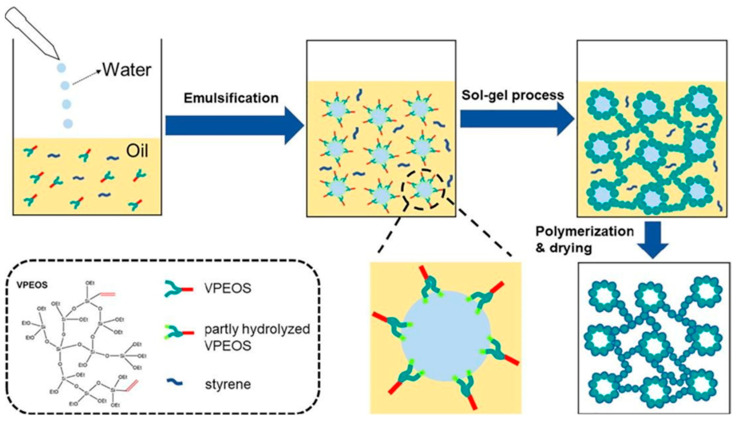
Polymer modification via polymerization of water-in-oil HIPE templates [17].

Different varieties of aerogels were produced during the last few decades as the methods for the synthesis and drying of aerogels improved. They can be classified as inorganic aerogels (silica, alumina, and titania), polymer-based, carbon allotropes (nanotubes and graphene), and natural macromolecule-based aerogels (alginate, starch, gelatin, protein, nanocellulose and chitosan) [13,14]. Typically, silica-based aerogels are the most potential candidate materials owing to their distinctive characteristics, such as low thermal conductivity (15–20 W/mK), low density (0.003–0.5 g/cm^3^), and large surface area [15]. They are generally fragile, have poor mechanical properties, and require a lengthy processing technique, hence limiting their application range [10]. Many attempts to increase the quality of silica-based aerogels have already been made, including using (i) adaptable silica catalysts in the strand, (ii) enhanced polymer cross-linking, (iii) accelerated ageing processes in different solutions, (iv) adding nanofillers, and (v) polymerizing the precursor in advance of gelation. For example, it has been shown that the combination of silica with methacrylate polymer to improve the polymerization resulted in enhanced mechanical performance and other parameters, including densities, areas, pore diameters, and void content [16]. Silica aerogels through polymer modification are illustrated in Figure 1. They are classified as silica aerogels reinforced polymer, fabricated via cross-linked via water-oil aqueous solution in high-internal stage emulsion substance. This novel material shows a superior performance property over pure silica aerogels [17]. In addition, Posada et al. produced ceria-containing silica aerogel via a three-way catalyst approach in incorporation with a new rapid supercritical separation method. They employed a polyether to strengthen the aging process and accelerate the gelation time [18]. This innovative technique can reduce the time taken to prepare wet gels, including gelation, ageing, and solvent exchange from days to seconds [19].

In addition, a nanofiller such as graphene nanoplatelets (GnPs) can also be employed to enhance the mechanical behavior of aerogel. This GnPs can speed up the gelatinization of nanostructures and reduce nanopore shrinkage throughout the hydrothermal process [20]. In addition, many studies are concentrating on improving the performance of silica aerogel by utilizing various approaches in native silica aerogel. A trifunction alorganoalkoxysilane, such as methyltrimethoxysilane was also used to provide agility to silica aerogel. However, the high costs of these precursors make them unsuitable for long-term use. As a result, many researchers adopted the organic-inorganic hybridization method, which entails cross-linking the silica aerogel with organic molecules [21]. This distinctive aerogel has a high degree of hydrophobicity and thermal insulation, giving it appealing properties such as self-cleaning, infrared stealth, and heat insulation compared with rival commercial items. The cellular structure shown the construction of multidimensional nanomaterials with synergistic action of organic–inorganic components contributed to the excellent multifunction of aerogel [22,23] and a strong interfacial effect is formed between the two components [24]. In general, other inorganic aerogels, such as alumina and titania, have garnered huge attention due to their unique microstructures. However, the extreme brittleness and manufacturing expense of these aerogels severely limit their industrial advantages. These aerogels may be modified with other materials, such as organic and polymer substances to provide numerous meshwork formation, high porosity, lightweight structure, moduli of elasticity, and low thermal conductivity [25,26,27]. Multifunctional inorganic aerogel with high open porosity and enormous surface area is a promising material that might be extended for extensive applications [28,29]. Additionally, the agglomeration of inorganic nanoparticles and nanofibers are recognized as a very viable approach for creating extremely flexible, readily accessible, and versatile composite aerogels [24].

Furthermore, polymer aerogels have a variety of forms, including polyamide (PI), polyvinylpolydimethylsiloxane (PDMS), and phenolic-based aerogels. All polymer aerogels have closely similar structures and properties [30,31,32]. In contrast to silica aerogel which are fragile and hygroscopic, aerogels derived from polymers have a broad variety of uses owing to their excellent mechanical attributes, such as high strength and fatigue resistance. These organic aerogels have thermal conductivity close to silica aerogel, comparable density and can be produced with very little shrinkage during the manufacturing process. Depending on the polymer type and fabrication circumstances, it may range between sheet-like skeletons and colloidal nanoparticles to nano/micro-fibrillar networks. The structural properties of aerogel materials, such as shape, size, and even pore ordering, have a substantial impact on their ultimate mechanical performance [33]. For example, a PI reinforced graphene oxide/cobalt (PI/rGO/Co) polymer produced by a unique cross-linking process demonstrated great heat stability and low thermal conductivity [34]. Additionally, multifunctional polyvinylpolydimethylsiloxane (PDMS)-based aerogels were reported to have high hydrophobicity and super-flexibility, thermal superinsulation, effective water, and oil separation, integrate selective absorption, and strain sensing [35]. In contrast, cellulose-based aerogel offer high porosity, higher surface area, and lightweight [36]. Aerogels containing organic precursors such as resorcinol formaldehyde, phenol formaldehyde, or melamine formaldehyde, on the other hand, have extremely poor electrical conductivities and dramatically lowered heat transmission throughout the aerogel’s backbone phase. Compared with cellulose-based aerogels, they may also be mechanically more flexible and confined to surface areas of less than 1000 m^2^/g [37].

**Figure 2:**
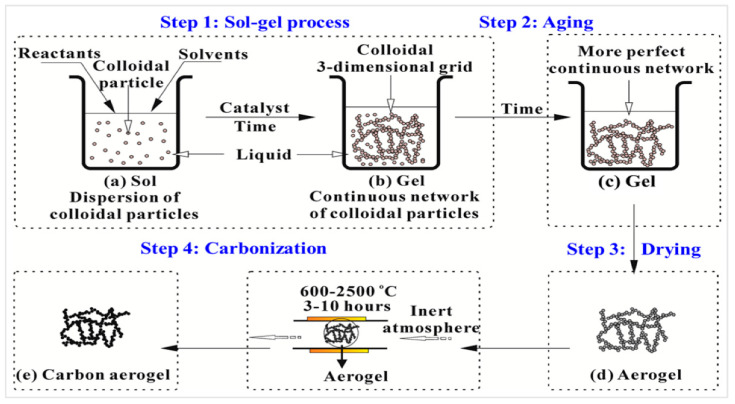
Basic method of producing carbon or polymer aerogels [33].

Meanwhile, carbon allotrope aerogel is generally porous materials made up of small interstitial pores (less than 50 nm) and interconnected with homogeneous carbonaceous particles (3 nm–30 nm) [38]. This aerogel has strong thermal and electrical conductivity. It provide a more brittle structure with higher backbone porosity due to micropore structures at specific areas of approximately 2000 m^2^/g for certain meso- and macrostructures [39]. The typical synthesis process of polymer or carbon aerogel is illustrated in Figure 2.

Macromolecules or polysaccharides-based aerogels are made from biopolymers derived from renewable raw materials such as cellulose, chitosan, alginate, chitin, and protein. For example, cellulose aerogel is identical to ordinary silica and polymeric aerogel in terms of compressive stress (5.2 kPa–16.67 MPa) and better recyclability [7]. As stated by Gong et al. the spongy morphology of this aerogel was steadily enhanced with the raising of the carboxyl proportion of nanofibrils in the structure. Carboxymethyl element could also effectively increase the total area of aerogel, due to the elimination of horrification [40]. Moreover, chitosan-based aerogel has much better physicochemical properties of the functional groups than cellulose-based aerogel and can be used in biomedical applications. When it was incorporated with graphene oxide, the adsorption capacity of this material improved [41]. In contrast, alginate-based aerogel is highly promising for low-flammability performance; however, it exhibits poor mechanical properties [42]. Interestingly, with the addition of graphene oxide, the catalytic property of this biomass aerogel can be increased by 30 times, resulting in an improvement in its mechanical property. The properties of different types of aerogels are shown in Table 1.

## 3. Synthesis and Preparation of Aerogels

**Figure 3:**
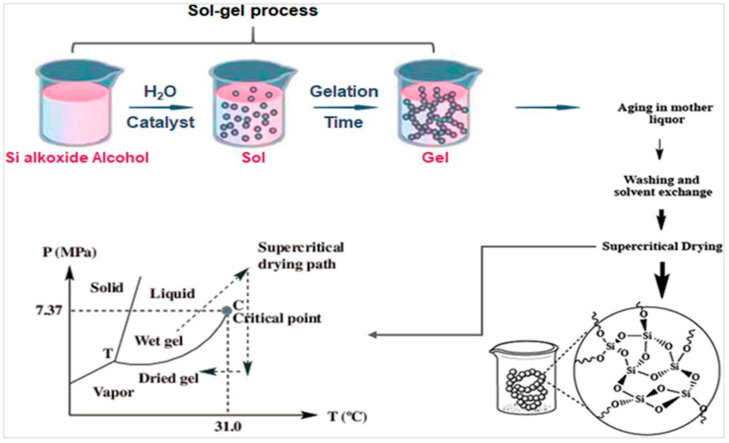
Schematic route of typical sol–gel synthesis [51].

An aerogel is often made via a procedure called the sol–gel technique [49]. Aerogel synthesis is a technique that is discussed by almost all types of aerogels. They are typically prepared in three stages: (1) sol–gel transition (gelation)/preparing the gel, (2) network perfection (ageing), and (3) gel–aerogel transition (drying) [49,50]. The formation of a three-dimensional (3D) network with high pore structure and proper strength is crucial in the preparation of aerogel structures. Figure 3 represents the general process of sol–gel synthesis.

(a) Sol–gel transition (gelation)

The sol–gel method is often used to obtain the gel phase. The fabrication technique begins with the creation of a colloidal dispersion, commonly referred to as a sol. Initially, the required antecedent is disseminated in a solvent. The insertion of a precursor then accelerates the gel-forming phase. This approach encourages polymerization via hydrolysis and gelation processes. The formation of a 3D nanopores inside a wet, gel-like substance is caused by cross-linking and splitting between polymeric components [51,52].

(b) Network perfection (ageing)

The resultant gel is subsequently treated in its stock solution to reinforce structural skeleton and strength. During ageing process, two separate mechanisms, namely Neck growth and Ostwald ripening, will act simultaneously to change the properties and structure of a gel at varying speeds. Throughout the procedure, material would be transferred to the neck, while colloidal particles can create a denser network.

(c) Gel-aerogel transition (drying)

The practice of eliminating most of the solvent from the gel is known as drying. During the drying cycle, the gel network cracks owing to capillary pressure created in the fine holes by the liquid-vapor interaction. Among the procedures described, drying the gel is by far the most crucial since it influences various aerogel properties [53]. The solvent, wastes, contaminants, and undissolved compounds should be eliminated, but the network structure should be left intact. The desired end-product characterization determines the drying process to be utilized. As an example, the most common techniques for curing wet gels are supercritical drying (at high and low temperatures), freeze drying, and ambient-conditioned evaporation [4,54]. Supercritical drying can preserve pores open and prevent them from collapsing. In contrast, the freeze-drying approach could produce aerogel with highly porous and minimal distortion. This method is known as simple, economically cost and environmentally friendly [55]. It retains the wet-gel rheology in solid state [56]. In addition, several drying techniques, such as ambient pressure, vacuum, and microwave, have now been tested in its formulation to cure the wet gel by liquid replenishment with air [57,58]. The ambient pressure drying process can generate numerous aerogels on an industrial scale, however it pollutes the environment, people, and animals due to solvent evaporation [59]. While microwave drying is unsuitable for commercial application because it generates porosities and induces aerogel molecules to dissolve. As a result, it reduces the surface area of the finished product.

The aerogels produced using the traditional sol–gel method have a monolithic structure. The conventional sol–gel technique in the fabrication of aerogels may be a one-, two-, or multi-step procedure based on the main precursor employed to form the aerogel. The above-mentioned approach is indeed the most often utilized way for manufacturing aerogels. For example, the sol–gel technique has been used to prepare chitosan–silica hybrid aerogel by mixing an inorganic network with an organic polymer [60]. The rough and irregular structure of the resulting chitosan from aerogel and the presence of a Si- O-Si polymeric network in the chitosan were obtained. Moreover, simultaneous sol–gel process combined with phase separation technique was developed for preparation of silica–polyvinyl alcohol (PVA) hybrid aerogel [49].

**Figure 4:**
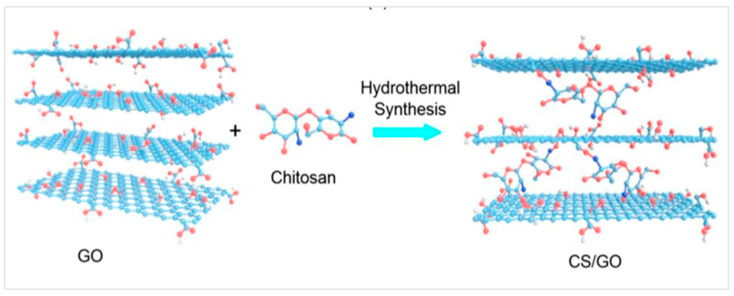
Schematic mechanism of graphene oxide with chitosan aerogel via hydrothermal method [63].

Other aerogel preparation methods are variations or derivatives of this procedure. Meanwhile, many applications such as drug delivery systems necessitate the use of microparticles with specific shapes [1]. To prepare these types of aerogels, new techniques have been used to modify the sol–gel process. Generally, cellulose aerogel is produced in three phases.: decomposing cellulose, generating cellulose gel using the sol–gel technique, then drying the gel to preserve its three-dimensional porosity nature [7]. Jiao et al. synthesized the cellulose aerogel according to the general methods but cooperated with hydrothermal procedure [61]. Meanwhile, Gupta et al. utilized the freeze-drying method to prepare cellulose aerogel with low thermal conductivity but high mechanical strength [62]. The hydrothermal approach has a commonly straightforward, high adsorption rate and requires no cross-linking reagent [63]. This method prevents the introduction of non-carbon contaminants, does not need binders, and is cost-effective. However, the use of energy to heat aqueous slurries has a very bad effect on the environment [60]. Figure 4 depicts the hydrothermal synthesis mechanism of graphene oxide aerogel.

More recently, through a sol–gel technique and freeze-drying, sodium alginate–silica aerogels with good absorption rate were produced from rice husk waste [64]. Moreover, the manufacturing processes largely impact the characteristics and prices of these aerogels. Additionally, sodium silicate and different silanes, such as tetraethoxysilane, polyethoxydisiloxane, and tetramethoxysilane, seem to be the most frequent catalyst used in the formation of silica aerogels [65,66]. Silanes are not commercially used to produce silica aerogels due to their higher cost and hazardous effects. However, for large scale manufacturing and practicability, sodium silicate is employed as a low-cost precursor in the synthesizing of silica aerogel [8].

Cross-linking agents such as glutaraldehyde, glyoxal (dialdehyde), ammonium persulfate (APS), sodium tripolyphosphate → butane→tetracarboxylic → acid → epichlorohydrin, and N,N′-metylenebisacrylamide are used in polymerization processes to create permanent bonds. In contrast, the weak mixture of hydrogen bonds, electrostatic pressure, and Van der Waals interactions are produced by physical cross-linking [67]. Pineli et al. created a graphene oxide/chitosan aerogel by cross-linking graphene oxide sheets via chitosan chains using APS. Electrostatic interactions between positive chitosan charges and negative functional groups of graphene oxide were observed to be increased [68]. Gong et al. also said that chemical cross-linking improved the mechanical performance of chitosan/graphene oxide aerogel [69].

## 4. Biomedical Applications of Aerogel

Aerogel is an appealing substance for the biomedical field due to their distinctive properties, which include low density, porous structure, extensive surface area, and high strength. It is also versatile in terms of sol–gel biochemistry. Aerogel typically used in biomedicine to encapsulate bioactive compounds with low solubility or stability as well as to create artificial scaffolds for tissue engineering and materials for chronic wound dressings. Other than that, many studies also discussed the applications of aerogel, such as for drug delivery carriers, anti-toxicity, and antioxidants. Although the technology and composition of aerogels are varied, the aerogels applied in the biological system must be made of biocompatible, and preferably biodegradable material.

Biocompatibility is the ability of materials to be functional in a biological system without causing harm. Biocompatibility must be determined before any substance can be used in biological applications. Table 2 summarises biocompatibility testing of various materials using in vitro and in vivo methods. Bajpai et al. studied the biocompatibility of 3D-structure graphene aerogel (GA). This 3D, ultra-lightweight and hydrophobic GA was produced by the one-step pyrolysis of sugar and ammonium chloride. GA showed excellent adsorption capacity for various biogenic amines, bacteria contaminants such as Staphylococcus aureus, and other toxins, especially in food safety applications. The biocompatibility of the synthesised GA is also determined by cell proliferation efficiency and wound healing ability [70]. In another work, Liu et al. created an extremely porous aerogel consisting of graphene oxide (GO) and Type I collagen (COL) using the sol–gel approach. This study demonstrated that 0.1% GO-COL aerogel had good biocompatibility in vivo, making it a potential scaffold to support bone regeneration and tissue engineering [71]. Zhou et al. found that the combination of hydroxyapatite and graphene to produce a new aerogel improved microbial electrocatalysis due to its higher interfacial and biocompatibility for bacterial growth [72].

Other biomaterials, such as bacterial cellulose (BC) also had excellent biocompatibility and had a low immunogenic potential [73]. BC aerogel is known for being fragile, very light, open-pored and transversally isotropic materials for various biomedical applications. Salehi et al. put clay nanoplatelets over the BC membrane to form a nano-fibrillated template for aniline in situ polymerization, creating a double linked network of electrically conductive pathways in the aerogel. Clay and polyaniline had a synergistic effect on biocompatibility and cell adhesion, with no mutagenic or carcinogenic effects [74]. In another study, a novel biocompatible BC aerogel modified with poly (glycidyl methacrylate) (PGMA) was fabricated. The incorporation of PGMA and BC aerogel improved its biocompatibility following the immobilisation of catalase [75]. BC aerogel had the highest modulus, porosity, and specific surface area among cellulose aerogels. Even so, the production of BC was hindered by a lengthy production period, a low yield, and a high price, which diminished interest in its further clinical applications.

Another biomaterial with exceptional properties and being more biocompatible within cells is silica aerogel. Their main limitations however, are fragility and high hygroscopicity [76]. In a study by Lazar et al., silica aerogel was hybridised with industrial manufactured bovine casein, using tetramethyl orthosilicate (TMOS) and co-gelation. The CHO-K1 Chinese hamster ovary cell line was used to test the in vitro biocompatibility of hybrid aerogel. It has been demonstrated that silica-casein aerogel are highly biocompatible and, to all intents and purposes, non-toxic to CHO-K1 cells [77]. According to the findings by Sani et al., the hydroxyapatite (HA)-mixed with silica aerogel with a weight ratio of 0.5 had the highest bioactivity and biocompatibility [78]. Resveratrol has been thought to help with or even cure osteoarthritis. Qin et al. synthesized a resveratrol-loaded silica aerogel (RSA) using the sol–gel method and exploited it as a drug delivery vehicle. According to the results of the study, RSA is inexpensive, biocompatible, and has relatively high loading rate of 19%. Initial in vitro toxicity testing revealed that RSA is biocompatible stable, and may be used to treat osteoarthritis due to its anti-inflammatory effects [79]. In another report, nanofibrous silica hybrid aerogel was biocompatible to healthy cells but their antitumour activity significantly increased when loaded with camptothecin (CPT) [80]. Kiraly et al. in their study injected a fluorescein-labeled silica-gelatin aerogel microparticles (FSGM) into the peritoneum of mice to assess acute toxicity. They reported no physiological abnormalities or disorder were discovered after a three-week-long experiment [81]. A nano-porous silica aerogel was developed for drug delivery for oral administration of paclitaxel (PTX), an anticancer drug. The excellent biocompatibility of this aerogel was proven by the reduced side effects of drug and inhibited tumour growth [82]. Furthermore, silica–gelatin hybrid aerogel has a potential for local and non-invasive drug delivery because they are biodegradable and biocompatible within tissue cells [83]. In addition, aerogels produce from marine polymer such as chitosan exhibit a potential prospect in wound healing due to their antimicrobial activity. Piatkowski et al. developed a new chitosan-based aerogel with enhanced properties to improve the healing of burn wounds. The studies demonstrated that the proposed chitosan aerogel containing Au nanoparticles were biocompatible and promoted fibroblast proliferation [84]. Batista et al. developed a hybrid alginate-chitosan aerogel fibre and assess their effect in wound healing application using the emulsion gelation method. In vitro model assessment revealed that they were non-cytotoxic and promoted wound healing [85]. In another study, the hybrid chitosan–alginate aerogel microparticles were also prepared using the emulsion gelation technique. The toxicity test showed that the alginate-chitosan carrier induced moderate lung inflammation along with some damage to kidneys and liver [86]. However, conventionally prepared chitosan aerogel exhibited a number of defects, including low porosity, an irregular structure, and an easiness to deform, which limited their biocompatibility [87].

Beside chitosan, alginate is another biomaterial that has been intensively researched in biomedical fields. For example, Franco et al. used mesoglycan (MSG) and impregnated calcium alginate aerogel (CAA) to treat a wound. Both human keratinocytes and fibroblasts were resistant to the cytotoxic effects of MSG on CAA, as shown by an in vitro experiment [88]. In other applications, aerogel microspheres based on alginate and hyaluronic acid demonstrate high porosity and good in vitro aerodynamic properties [89]. Carbon-based aerogels are unique since it consists of networks of 3D nanostructures with a high volume of air-filled nano-porous, high porosity, low density, and a large surface area [90]. These properties endow aerogels with a rapid response signal, high selectivity, and super sensitivity for sensing a variety of biomedical targets. The synthesis of carbon-aerogel scaffolds containing biocompatible ceramic nanoparticles of tricalcium phosphate has been disclosed by Tevlek et al. Due to their high gelatine content and highly porous structure, the materials exhibited good biocompatibility and supported cell growth for 14 days [91].

**Table 2:** Summary of studies on compatibility of aerogels for biomedical applications.

	Aerogels	Method	Remarks	References	Year
1	Cellulose	Freeze drying and polymerization	Higher biocompatible with catalase immobilization	[75]	2019
2	Silica	Freeze-drying	Biocompatible for drug carrier	[82]	2019
3	Graphene oxide- collagen	Sol–gel process	0.1% GO-COL aerogel presented reliable biocompatibility	[71]	2019
4	Graphene	Pyrolysis	Cell viability was observed even at high concentrations	[70]	2019
5	Chitin	Supercritical CO2 drying and freeze-drying	Good biocompatibility (cell viability >90%	[92]	2019
6	Alginate- chitosan	Supercritical drying of CO_2_	Cell viability values >70 %	[85]	2020
7	Alginate- Chitosan	Emulsion gelation	Resulted in mild lung- congestion	[86]	2020
8	Silica	Aqueous sol–gel ambient pressure drying	Not toxic to normal human osteoblast cell line	[78]	2020
9	Silica	Co-gelation in the sol–gel, supercritical CO_2_	Highly biocompatible and practically inert towards CHO-K1 cells	[77]	2020
10	Silica	Sol–gel	Good biocompatibility	[79]	2020
11	Silica	Freeze-drying and cross- linking	Excellent biocompatibility to human cells	[80]	2020
12	Carbon	Freeze-drying	Cells able to adapt to microenvironment and able for growth	[91]	2020
13	Composite	Freeze-drying	Good biocompatibility of mouse lung fibroblasts (L929) cells on the membrane	[74]	2021
14	Silica	Sol–gel combined with co-gelation	All mice were healthy after being injected with aerogel	[81]	2021
15	Graphene	Hydrothermal thermal dialysis and freeze-drying	Excellent biocompatibility	[72]	2021
16	Chitin	Supercritical CO_2_ drying	Lower haemolysis ratio (<1%)	[93]	2019
17	Alginate	Supercritical CO_2_ drying	Not cytotoxic	[88]	2020
18	Cellulose	Supercritical CO_2_ drying	Excellent conditions for cell viability and proliferation	[94]	2021
19	Magnetic	Sol–gel	Biocompatible	[95]	2022
20	Silica	Sol–gel, supercritical drying	Biocompatible for local and non- invasive drug delivery	[83]	2022

### 4.1. Drug Delivery Carriers

Scientists have built a variety of organic and inorganic substances that can be employed as drug-delivery vehicles. These materials can improve the safety and effectiveness of drugs by making them more stable, soluble, and long-lasting. Xie et al. examined 3D porous silica aerogel as carriers for antibacterial compound release, specifically cinnamaldehyde, salicylic acid, and sorbic acid. They discovered that the aerogel inhibited *Escherichia coli* (*E. coli*) during the initial stage; interestingly, they were still efficient when 90% of the cinnamaldehyde was dissipated [96]. They came to the conclusion that mesoporous silica aerogel is an interesting antibacterial vehicle. In another study, the chitosan-reinforced vancomycin aerogel was investigated as a potential treatment and prevention compound for surgical site infections. Release profiles from aerogel carriers and vancomycin content revealed a rapid drug release, allowing for efficient local therapeutic doses to be achieved [97]. Furthermore, vancomycin particles were also demonstrated to be efficient in lowering excessive bacterial loads at the wound site along with cytocompatibility.

Gorshkova et al. produced alginate–chitosan aerogel by combination of sol–gel and supercritical drying technologies. This aerogel demonstrated prolonged levomycetin release rates and has the potential to be employed in a drug delivery application [98]. Simonson et al. investigated a gel-like aerogel in finding the weakness of *Mycobacterium tuberculosis* (MTB) and tuberculosis (TB)-infected cells to provide correlated pair of peptide and antibiotic for strong and quick antituberculosis treatment. The aerogel could be used as a versatile inhalable substrate for the development of innovative biomaterials-enabled therapies for pulmonary multidrug-resistant (MDR) TB by using hyaluronic acid (HA) as a key component in the aerogel structure [99].

### 4.2. Polysaccharide/Chitosan Aerogel for Wound Healing

**Figure 5:**
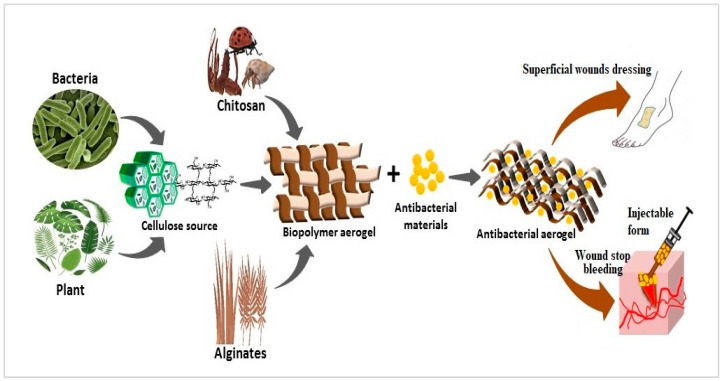
Schematic drawing of biopolymer aerogels for wound healing [100].

The porosity of aerogels enables them to absorb a greater volume of secretions at wound site. This, in turn, will reduce inflammation and prevent bacterial infections from developing in the wounds. Because of their low toxicity, high stability, and non-allergenicity with good biological performance, aerogels from polysaccharide are also often used in wound care. Polysaccharide aerogel’s solid structure expands and inhibits the growth medium for living cells. They too can contain a primary active substance such as an antimicrobial drug, to help and speed up the healing process [48]. Figure 5 shows the schematic drawing for biopolymer aerogels in wound healing applications.

The use of chitosan-based aerogel as antimicrobial agent could give rise to immunogenicity. Chitosan performed better when combined with other materials as a possible antimicrobial wound treatment. The most of chitosan’s antimicrobial effect is related to the fatality damage of intracellular substance resulting from cell membrane failure and changed penetrability [101]. Zhang et al. developed quaternised chitosan/PVA-polycaprolactone/curcumin (QCS/PVA-PCL/Cur) Janus aerogel for treatment of diabetes patients. The cellular structure and micropattern of PCL/Cur nanofiber observed contains a large volume of wound exudate that can be quickly drained, and effectively prevented from being absorbed back into the wound site. Therefore, janus nanofibrous aerogel can reduce inflammation while promoting diabetic wound healing [102]. Guo et al. obtained high porous structure and rigid aerogel in healing treatment from chitin nanoparticles. This chitin aerogel resulted in amazing capability to speed up the tissue repair because of their high functionalities from interlinked porosity, and improved hydrophilicity properties [92]. Whereas Batista et al. developed a novel preparation of alginate-chitosan aerogel fibres, evaluated the ability for wound closure in an in vitro study by scratch assay and evaluated their antibacterial activity. The study reported that hybrid aerogel had ability to close a wound and had antibacterial activity against *S. aureus* and *K. pneumoniae* [85]. More recently, Yan et al. discovered oxidized Bletilla polysaccharide Schift Base (ORBPS)/PVA aerogel from combination of freeze-drying and crosslinking demonstrated good antibacterial as well as hemostatic properties for wound healing [103].

Bacterial cellulose (BC) possesses a number of biorelevant properties, such as high absorption capacity, superior mechanical potency and biological compatibility. As a result, BC served as a matrix provider for enzymatic reactions, molecules, and treatments, especially in reproductive and antimicrobial properties. Revin et al. found that aerogel were long-lasting and had less delamination properties after prepared with TEMPO oxidized bacterial cellulose (OBC) compared with native BC (NBC). They also stated either NBC or OBC and sodium fusidate caused aerogel to have strong antibacterial effect on S. aureus [104]. Moreover, Lin et al. proved the high content of chitosan exhibited high antibacterial effects of the composite aerogel [105]. More recently, Chen et al. prepared antibacterial aerogel pads containing liposome- copper nanoparticles (CD/Lip-CuNPs) by using chitosan that was reacted with dialdehyde starch. The aerogel could freshly keep the fresh pork for two weeks [106]. Obviously, alginate is the most explored polysaccharides due to its outstanding characteristics including gelation, biocompatibility and biodegradability in medicine, and a food additive [107]. A composite material from alginate and chitosan was synthesized according to Pang et al. They also proved the antimicrobial properties of this material was significant for *S. aureus* and *E. coli* [108]. Jia and Wang fabricated three-dimensional nanofiber aerogel from methoxy polyethylene glycolpolycaprolactone for high water absorption capacity. The study confirmed the aerogel suitable as a carrier of antimicrobial agents [109]. In another study, a chemical-free approach was utilized to fabricate cellulose nanofibers (CNFs) and chitosan blended aerogel. The found the addition of chitosan considerably decreased absorption of water and allowed the aerogel to function better as antibacterial materials [110]. Table 3 summarised the wound healing and antimicrobial activity of aerogels.

### 4.3. Anti-Toxicity

Chitosan nanostructures have become a popular issue in biomedicine due to their antimicrobial properties, as well as their biocompatibility and high biodegradability without causing toxicity [101]. According to Piatwoski et al., chitosan aerogel doped with gold nanoparticles had the excellent swelling capability in an aqueous solution and were non-toxic when tested with L929 mouse fibroblasts. These biocompatible porous aerogel was shown to encourage fibroblast growth and can be used in tissue engineering [84]. Moreover, chitosan aerogel prepared by non-acid condition method shows the potential to serve as a wound- healing matrix, exhibits good swelling performance, and is non-toxic [101,115]. Furthermore, graphene oxide and chitosan aerogels reinforced by using a mix of seed and skin extracts from 200 País grapes were developed by Figueroa et al. They mentioned that this hybrid aerogel, under alkaline conditions, were not cytotoxic to human dermal fibroblasts [116]. Borges-Vilches J. et al. also did something similar. In their studies, gelatine and graphene oxide were blended with grape skin by using microwave-assited reactions. All aerogels suggested non-toxic impacts on human dermal fibroblast (HDF) cells [117]. In a recent study, Wu et al. evaluated a composite aerogel synthesised from chitosan and aramid nanofiber by using chemical cross-linking of glutaraldehyde for tissue engineering application. These materials found to be harmless and strongly promoted proliferation of cells [118]. Also, Zhang et al. successfully constructed aerogel from chitosan contained molybdenum disulphide nanosheets (NMN) via modified-amino and physical approach. The aerogel with 4 mg mL−1 NMN had resulted low toxicity in certain organs [87]. In another report, deacetylated derivative chitosan called chitin was synthesised by Song et al. to develop a chitin/graphite oxide aerogel. They obtained a lower haemolytic phenomenon indicated their safety and nontoxicity [93].

In addition, a novel resveratrol-loaded silica aerogel (RSA) was formulated by Qin et al. from the sol–gel method. Introduction of RSA in-vitro study had shown no cytotoxicity [79]. Furthermore, the methotrexate functionalised silica-gelatine hybrid aerogel (SGM) was also synthesised by Nagy et al. via the sol–gel and co-gelation method. The non-cytotoxicity effect of this SGM towards various cancerous and non-cancerous cell lines, which correlated with the collagenase activities of cells, was observed [119]. Tiryaki et al. regenerated the aerogel from silica and then coated them with hybrid inorganic silica aerogel nanoparticles. All the samples with absence and presence of amino and Dextran compound show no significant cytotoxic effect on colorectal adenocarcinoma cell lines (Caco-2 cells) [23]. Micro-spherical alginate-chitosan aerogel in the absence of crosslinkers was intentionally studied by Alsmadi et al. for use in lung cancer applications. Loading cisplatin on the alginate-chitosan aerogel carrier can reduce the harmful effect of lung however, rise up liver intoxication following intratracheal introduction with nephrotoxicity [120]. Moreover, Egu et al. proposed a similar work by using silica–gelatin hybrid aerogel reinforced with cisplatin as a drug carrier mechanism for cervical cancer chemotherapy. In this study, 1 mg/mL of hybrid aerogel show same antiproliferative properties with 0.5 µg/mL free dose cisplatin in cytotoxicity study [83]. Wu et al. produced an alginate aerogel that contained tigecycline (TGC) and octahedral copper crystal for antibacterial and local infection therapeutic. It was shown this type of aerogel had minimal biological toxicity and would be suitable for future use in bone tissue engineering [121].

**Figure 6:**
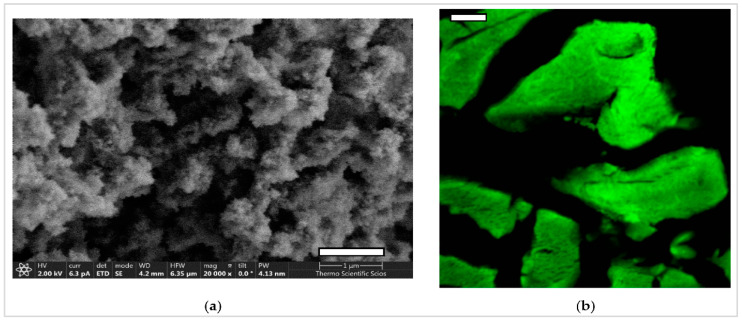
(**a**) SEM image of silica–gelatin aerogel hybrid and (**b**) fluorescence images of paraffin-encapsulated aerogel particles [81].

In addition, Kiraly et al. generated a hybrid silica-gelatin aerogel by combining the sol–gel and co-gelation procedures. This mesoporous aerogel exhibited minimal toxicity after being infused in mice’s abdominal cavity and placed in parathymic lymph nodes as shown in Figure 6 [81].

In addition, carboxymethyl cellulose/poly (N-isopropyl acrylamide) was also used as a functional group in novel hydrogel development. It was integrated in a drug delivery system after being integrated into polymer network of the aerogel. This responsive aerogel showed that after 24 h, the survival rate of NIH3T3 culturing cells was still above 90%. Therefore, this aerogel drug carrier had no obvious toxicity [122]. Moreover, the non-toxic nature of some materials such as graphene was also promoted by Shukla et al. to be used as part of the synthesized aerogel. Then, the toxic resistance of this material was proved to be non-toxic when it was employed at a concentration of greater than 20 mg/mL-1 for both cells. The synthesized aerogel was found to be completely safe for daily usage [111]. Hu et al. created an aerogel by from salecan and cationic starch (SAC). The performance of the developed aerogel was tested as biocompatible material since there were non-cytotoxicity and safety results from the studies [123]. This also same as aerogel derived from tricalcium phosphate (-TCP) indicated it had no in-vitro cytotoxicity activities [91]. In addition, a stable magnetite hydrosol was fabricated by modifying the sol–gel transition by varying concentrations and drying conditions to achieve stable functional properties of the aerogel. The porosity of this monotonic aerogel affected the expression of its hemostatic function, improved blood absorption, and generated mild cytotoxic effects at concentrations up to 1 µg/mL [95]. Table 4 lists the anti-toxicities activities of different types of aerogels.

### 4.4. Antioxidant

Antioxidants able to inhibit reactive oxygen molecules produced throughout the aerobic cellular metabolism [138]. In other words, it is considered as a molecule which hinders the oxidation of molecules within a cell. Free radicals are unstable since they able to initiate chemical reaction, leading to injuries or cellular fatality. Therefore, antioxidants have the ability to stop the reaction by removing these free radicals intermediate [139]. Aerogel become one of the significant substances with antioxidant properties in bioactive encapsulation and controlled release, food conservation and packaging, and moisture regulator, as shown in Table 5. Pectin, also known as soluble fibre, is a broad class of long polysaccharides that are linked together to form α segment of neutral sugars. Due to the obvious availability of fruits such as pear, banana, corn, bean and citrus, it has been confirmed to be reliable, healthy, sustainable, and affordable. Chen et al. successfully prepared the stable aerogel from pectin cooperated with alginate to control proanthocyanidins withdrawal from the aerogel matrix. This novel carrier, which comprised pectin-rich aerogel microspheres, showed greater antioxidant activity as well as adequate polyphenol encapsulation [126]. More recently, Wu et al. developed an aerogel reinforced with citrus pectin and cellulose nanofibre to resist edible fungus in food packaging applications. The study mentioned that the total antioxidant capacity of Agaricus bisporus (mushroom) was preserved, and the fresh-keeping period was increased to five days [136]. The incorporation of phenolic-rich natural extracts into aerogel is an appealing strategy for the release of bioactive chemicals, which are often used as antioxidants in food industries. Their antioxidant activities could impact agricultural consumer goods and improve food storage stability. The addition of antioxidant substances in the aerogel might minimize the potential of meat to decolorized due to oxidation of the molecules inside the cells. For example, Fonseca et al. investigated the possible use of starches derived from maize filled with pinhao coat extract (PCE) as phenolic chemical carriers in food packaging applications. It was obtained that huge quantity of phenolic compound liberated from hybrid aerogel with low density and high-water absorption properties. Additionally, the powerful antioxidant effect at 10 % of PCE was observed [133]. Moreover, Vigano et al. produced alginate aerogel which consisted of phenolic extract obtained from passion fruit bagasse and gallic acid (GA). However, it discovered that the wet impregnation and supercritical drying techniques used to generate these active chemical aerogel had no effect on the antioxidant properties [130]. Cellulose and nanocellulose are interesting polysaccharides which exhibit properties such as biodegradability and non-toxicity [140]. Lignocellulosic materials perhaps acquired from algae, agricultural and wood side-product. However, extraction from abandoned products which do not play important role in the food chain are advantageous to discover. For instance, the effectiveness of cellulose-based aerogel, which consisted of A. donax waste biomass in the antioxidant activities was evaluated by Fontes-Candia et al. These superabsorbent bioactive aerogels were formulated to significantly offered the highest antioxidant capacity from biodegradation and colour depletion throughout packing of mashed red meat after 10 days [127]. In another case, the antioxidant capacity test was constructed for aerogel studied by Benito-González et al. for meat preservation. They discovered that the aerogel had a good inhibitory effect (23–91%) and potentially to be good packaging materials as able to prevent oxidation of red meat in cold storage [131]. One of the challenges connected with the creation of cellulose aerogels is that often they are produced through a complicated synthesis approach, which comprise many processes and ultimately increase the processing costs. Such procedures are inappropriate for food-products due to chemicals usage during production. Because cellulose-based aerogels have a lot of internal wall area contributed for large surface area, they are an excellent choice for bioactive ingredient for drug delivery and pharmaceuticals. In another work, Zhang et al. fabricated composite aerogel from bamboo shoot and sodium alginate. The absorption of curcumin (used as a drug model) was decreased by these porous cellulose spheres. They proved that the non-crossed composite aerogel with Ca^2+^ had higher adsorption capacity and excellent outcomes in antioxidant analyses [132]. Also, in biomedical field, the cellulose from janus nanofibrous was used as a bioactive element in wound healing to speed up the healing time [102]. Cellulose and nanocellulose aerogels as bioactive delivery vehicles from yerba-mate (*Illex paraguariensis*) were produced through valorisation. Both aerogels demostrated great performance by retaining yerba-mate based extract (YMBE)’s possibility to sequester the DPPH and were suitable for food packaging [128]. In another case, the valorisation used for production of bioactive aerogel from Gelidium sesquipedale seaweed in food packing was explored by de Oliveira J.P. The produced hybrid aerogel contained agar-based extract consisted of polyphenol and presented antioxidant capacity [125].

In addition, it was worth to note that the excellent properties of chitosan causing it widely used as precious material to lower the oxidant properties of the other materials. Due to this reason, Radwan-Pragłowska et al. synthesized a hybridized cellulosic of *Tilia platyphyllos* with chitosan to fabricate a novel aerogel material under microwave reactor- assisted conditions. The antioxidant activity was significantly increased after the addition of the synthesized aerogel resulted from the presence of the phenolic compounds [124]. Further, Coldebella et al. produced aerogel via combination of nanofibrous cellulose (NFC) from Tajuva and Eucalyptus trees, with sodium alginate by the co-grinding process. This material was able to absorb a high amount of water owing to the high antioxidant capacity attributed to the existence of phenolic and tannin substances [134]. Cellulose plant derivatives generally have a great potential for usage in hybridized aerogel as a cosmetic, nutraceutical, and pharmaceutical element. However, additional in-depth study, such as clinical in vivo trials, is advised to even further assess these highlighted characteristics. Furthermore, caffeic acid (CA) belongs to the hydroxycinnamic acid group. CA has great antioxidant effects and is consequently used in several dietary supplements to enhance athletic performance, reduce exercise-induced weariness, promote weight reduction, and treat cancer. The excellent antioxidant properties of CA have also employed in aerogel in many supplementary foods. In recent study, Thongchai et al. studied effectiveness of CA incorporated with chitosan and collagen aerogels. The results proved that these composite materials suitable for medicinal and aesthetic applications [129]. Moreover, chitosan is one of the most desirable biomaterials for pharmaceuticals and dietary supplements. It is one of the two most common naturally produced biomass such as shrimps and other crustacean shells. Chitosan is characterized structurally as a linear polysaccharide. Lin et al. found that chitosan aerogel composite films had strong antibacterial activity. However, a weak antioxidant response was found at a lower amount of chitosan and nano silicon aerogel [105]. In other work, Zhu et al. established attapulgite-based silk fibroin composite aerogel (ASA). They concluded ASA preserved their structure and antioxidant effect of polysaccharides [135]. Aerogels’ specific surface area and high porous structure (interconnected mesopores) enable more rapid depositing of small-molecule drugs, finite access to innermost regions of the matrix, and optimum interactions of biological milieu with the polymer matrix when compared to other 3D materials [141].

### 4.5. Bone Regenerative

**Figure 7:**
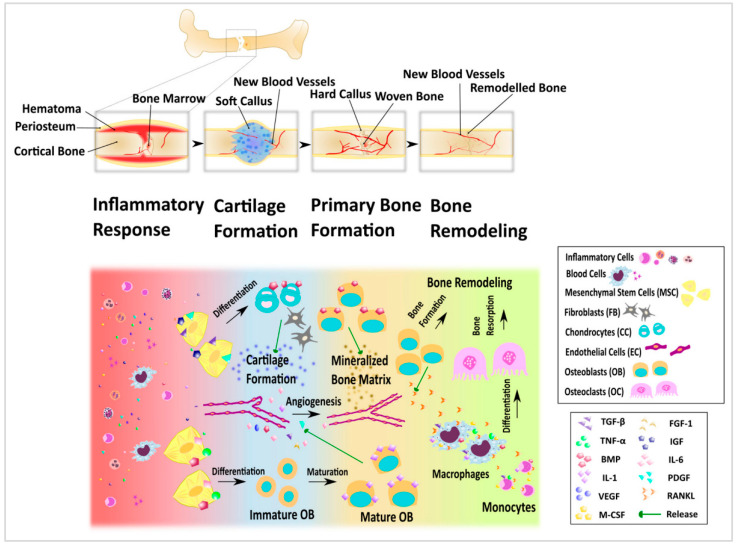
Phases for of bone breakage healing [143].

The development of biocompatible porous supports holds great potential for the regeneration of severely damaged bone tissues [142]. Bones are formed from a dry weight composite of 30% organics (collagen and glycoproteins) and 70% minerals (mostly hydroxyapatite (HA) nanocrystals. Bone tissue needs both the inorganic HA and the organic Type I collagen. Lifelong growth, repair, and renewal of bone tissue are mediated by anabolic osteoblasts, catabolic osteoclasts, and osteocyte signaling as in Figure 7 [143].

Nanosized HA crystals and connective collagen fibrils make up the bone biomineralization activities [144]. Cancellous bone is made up of a mineralized organic matrix network with a 40–90% porous structure. Some aerogel-based biomaterials have 3D cross-linking systems that resemble the molecular structure of cancellous bone [145]. Huang et al. made an aerogel from ultralong HA nanowires with ultrahigh porosity (98.5%) that is elastic and highly porous. They also demonstrated that the HA nanowire aerogel scaffold was conducive to the ingrowth of new bone and blood vessels, thereby accelerating bone regeneration and neovascularization to a remarkable degree [146].

Biopolymer-based scaffolds were employed in bone regeneration because of their degradability and high compatibility, which are two vital properties in bone tissue engineering scaffolds [147]. Maliki et al. developed a hybrid aerogel consisting of silica and silk fibroin hybrids, resulting in the honeycomb-shaped and lightest porous structures. The research found that the silica–silk fibroin aerogel was a very excellent scaffold for osteogenesis development. In addition, an in-vivo study revealed the creation of new bone tissue at the defect location after 25 days post-implant [148]. Goimil et al. found that submicron-sized silk fibroin aerogel, combined with poly (ε-caprolactone) (PCL) and dexamethasone also promoted bone repair in another study [149]. Cellulose nanocrystals (CNC) may be utilized to phosphorylate various cellulose substrates (such as bacterial and nanofibrillar cellulose), as well as induce HA development, which is an important indication of osteogenesis [150]. Kamel et al. created 3D aerogel implants by combining sugarcane pulp cellulose synthesis using TEMPO-oxidized nanofibrillated (TONFC) and combining it with glucosamine hydrochloric acid. It was then infused with rosuvastatin and strontium borate. The study showed that TONFC formulated with rosuvastatin, with the help of glucosamine and strontium borate, could be used for dental socket preservation [151,152,153].

In another study, β-Tricalcium phosphate and silica were mixed to create β-TCP-silica aerogel, which were then evaluated for bone tissue regeneration. It found that the aerogel composites treated at temperatures around 800 °C had a positive effect on MG63 cells osteogenic activity [154]. Natural polymer aerogel mechanical properties are extremely poor for large bone repair. Aerogel will be stronger and has better mechanical properties if they have inorganic fillers such as HA, silicon dioxide (SiO_2_), and graphene added to them [152,153]. HA is a well-known one of the essential minerals of human bone [155]. Incorporation of HA into an aerogel compatible with bone enhanced its mechanical strength [156]. Iglesias-Mejuto and Garcia-Gonzales have recently investigated the use of reinforced alginate-HA aerogel scaffolds in bone tissue engineering. An optimized scaffold formulation obtained by dual cross-linking with calcium chloride (CaCl_2_) and glutaraldehyde provided long-term stability and had the bioactivity needed for bone regeneration [157]. Whereas the composite aerogel of silk fibroin/cellulose aerogel reinforced by nanohydroxyapatite filler has the same mechanical strength as cancellous bone at an optimal ratio and exhibited higher cell proliferation and differentiation capacity [158].

More recently, Liu et al. proved that blending SiO_2_ nanofibres into super-elastic organic/inorganic composite aerogel gave it good mechanical properties and elasticity. Furthermore, the induced SiO_2_ with aerogel demonstrated excellent potential for bone regeneration, including osteogenesis and angiogenesis [159]. Graphene oxide (GO) also has excellent mechanical and biocompatibility properties, making it an ideal component in the development of hybrid aerogel for bone regeneration [160,161,162]. The compressive strength of the structure and its interaction with the surrounding tissues are both affected by the pore size in the GO aerogel [142]. GO composite made from GO and nano-HA (nHA/rGO) was used to make a biomimetic scaffold for repairing bone defects. The study found that 20% nHA/rGO scaffold could promote wound healing in six weeks [163]. The presence of more functional groups, which aid in mineralization and the growth of apatite, was observed in polymers such as collagen and chitosan, as well as in functionalized reduced GO aerogel [164]. Asha et al. found that reduced GO aerogel functionalized with chitosan accelerated the mineralization of HA. These mineralized aerogel mimicking natural bone had higher rates of cell proliferation, osteogenic differentiation, and osteoid matrix [165]. In other case, Bahrami et al. shown that decreasing the amount of GO covering collagen increased the viability and proliferation of human-bone-marrow-derived mesenchymal stem cells (hBMSCs) [166].

### 4.6. Cartilage Tissue Repair

Cartilage has many features, such as being able to resist compression forces, make bones more flexible, support skeletal areas, and be flexible when needed. The chondrocyte is the main cell that tends to make cartilage. It lives in the lacunae [167,168]. Since cartilage tissue lacks blood arteries and nerves, its ability to self-heal is restricted [169]. Cartilage scaffolds fabricated from aerogels need to be good for their biocompatibility, good renewability, little immunological impact, favorable cellular contact, and geometries equivalent to the Type II collagen structure in the cartilage matrix [170]. Therefore, aerogels made from natural polymers have been widely employed in bioengineering for tissue regeneration. These aerogels have been proven to offer an adequate bioactive microenvironment and strength to promote the development of new chondral muscles at damaged locations [171,172].

Intriguingly, electrospinning is recognized as a flexible method for producing nanofiber aerogel with diverse architectures for many purposes, including tissue engineering. This technique has enormous potential as a technology for generating 3D aerogels and scaffolds with high reliability [173,174]. modified a framework of electrospinning gelatin/PLA nanofibers cross-linked with hyaluronic acid, resulting in facilitated cartilage healing [175]. Three-dimensional printing and nanofiber diffusion of poly(l-lactide)/gelatin-aerogel might provide tracheal constructions with a biomimetic extracellular matrix (ECM)-like morphology for tissue repair [176]. In other research, gelatin polycaprolactone (GT/PCL) nanofiber aerogel coupled with an ECM scaffold using an electrospun technique exhibited a tissue architecture comparable to those of real cartilage. It created a stable microenvironment for chondrocyte attachment, stimulated collagen formation, and hence expedited cartilage damage recovery [177]. Furthermore, gradient fibrous aerogel composed of glycosaminoglycan and biomineralized fibers grafted with chemokine peptide (E7) led to the repair of osteochondral interfacial tissue [178]. Hong et al. injected a super elastic poly (dimethyl siloxane) (PDMS) into graphene to build excellent stress-transfer 3D graphene aerogel. This aerogel displayed complete reversible structural deformations and excellent compressive strength, making them appropriate for cartilage repair [179]. Numerous efforts have been made to employ collagen in association with proteins or other biopolymers to replicate synthetic tissue [180]. Different collagen-based composites made by integrating natural/artificial polymers and bioactive inorganic compounds were distinguished by their porosity structure and ability to enhance cell adhesion. These collagen matrix aerogels increased the mechanical strength, structural stability, and osteoblastic activities of cartilage regeneration [181,182]. Munoz Ruiz et al. revealed that a collagen-alginate-GO aerogel scaffold had a porous structure with a nonporous external wall. This scaffold facilitated cell adhesion and proliferation when osteoblasts were embedded on scaffold surfaces [183].

**Figure 8:**
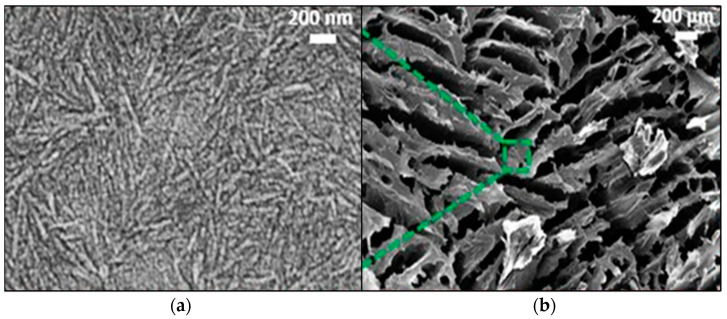
Scanning electron microscopy (SEM) image for (**a**) aerogel structure and (**b**) cellulose nanocrystal (CNC) constituted of the aerogels [187].

Moreover, polysaccharides, which have structural similarities to the ECM, might offer an alternate aerogel for native cells in cartilage regeneration. In some cases, the properties of aerogel scaffolds can be altered by mixing nano cellulose due to their renewability, hydrophilicity, and excellent strength [184]. For example, Mirtaghavi et al. created 3D-structured nanocellulose-based aerogel with highly porous and interlinked pores that is suited for biological applications [185]. Tang et al. used stereolithography and freeze-drying to fabricate aerogel based on polyethylene glycol diacrylate (PEGDA) reinforced cellulose nanofibers (CNFs) for cartilage tissue repair. It resulted in a negative Poisson’s ratio scaffolds in the growing microenvironment for stem cell proliferation [186]. Additionally, Li et al. explored the 3D direct ink write (DIW) approach for printing pure cellulose nanocrystal (CNC) aerogel for cartilage tissue repair. Figure 8 shows this 3D structure with a special inner pore design permitted the creation of a CNC aerogel scaffold tailored to cell integration needs [187].

### 4.7. Dental

Aerogel can be one of the most practical sources in dental applications due biocompatibility and non-toxic properties [188]. Aerogel able to cooperate with other materials in production of composite with highest porosity, lowest thermal shrinkage, and good mechanical material. Silica aerogel, the most sophisticated aerogel substance, had great economic health implications. Silica aerogels have high potential in dental areas. The aerogel’s large specific surface area, which is utilised to prepare dental compounds with inorganic fillers. As reported by Aminoaraya et al., they used silica aerogel as a reinforcement filler. This study enhanced the mechanical properties of dental composites [189]. Moreover, silica aerogel plus resin composites were effectively created by mixing the filler material in restorative dentistry with a resin matrix. They discovered that the silica aerogel’s existence gave the composite its exceptional antibacterial qualities, satisfying the need for antimicrobial features in dental composites [190]. Moreover, the same filler material was combined with a polymer known as poly(methyl methacrylate). The presence of silica aerogel strengthened, enhanced brittleness, and improved impact durability of the composite materials [191]. Amiri et al. manufactured dental composite made of triethylene glycol and bisphenol A-glycidyl methacrylate (Bis-GMA/TEGDMA) and filled with hydrophobic silica aerogel. Using an aerogel filler improves the composite’s whiteness index when compared to primary resin, increased compressive strength and proportion of cells survived while lowering the toxicity of the finished dental composite [192]. In other study by Lazar et al., they produced biomaterials from mesoporous silica using the sol–gel technique. These substances, together with hydroxyapatite and -tricalcium phosphate, operated as bioactive agents and may be used in dentistry as potential replacement materials for sick or impaired bone tissues [193].

Moreover, silica aerogel as bioactive ceramic manufactured with β-tricalcium phosphate. This product seemed to be applicable in the case of a novel scaffold ceramic for dentistry [194]. In other case, a 3D aerogel implant composed of nanofibrillated cellulose loaded with rosuvastatinthe designed in combination with strontium borate can be used safely in dentistry for dental socket reservation as studied by Kamel et al. [151].

## 5. Coronavirus Disease (COVID-19)

**Figure 9:**
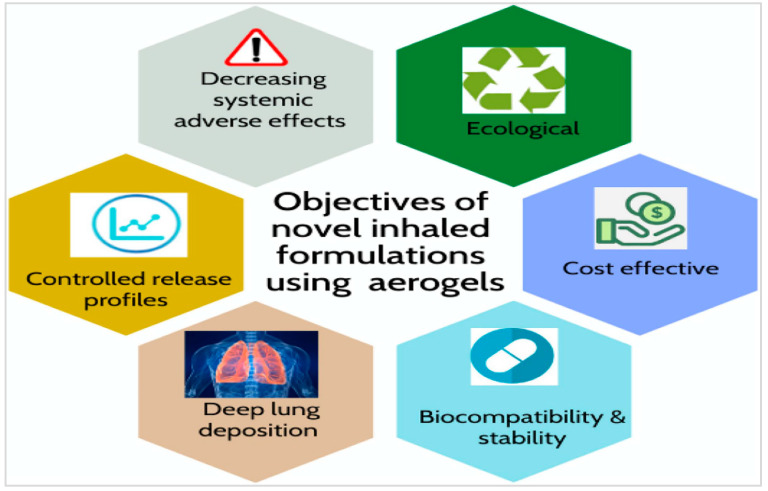
Possible advantages of aerogel-based carriers for the administration of pulmonary drugs [195].

The World Health Organization (WHO) declared the coronavirus infection (COVID-19) to be an outbreak on 11 March 2020. Although some studies have shown that aerogel may be used to treat patients successfully, there is no widely accepted cure for this fast-spreading illness. The present COVID-19 epidemic has sparked a resurgence of interest in pulmonary medicine drug carriers and their use in the treatment of respiratory illnesses. For example, according to Duong et al., for the existing COVID-19 condition, aerogel technologies have great potential for resolving pulmonary drug-delivery problems as in Figure 9 [195].

In addition, Azithromycin (AZM) or hydroxychloroquine (HCQ) embedded in graphene-aerogel was used as a potential therapy to treat COVID-19 by Mater Mahnashi et al. A simple, rapid, economical, and sensitive electrochemical sensor was proposed for the simultaneous analysis of HCQ and AZM. The sensor was made of modified GCE with vanadium disulfide quantum dots decorated nitrogen and sulphur co-doped graphene aerogel/carboxylated carbon nanotubes (VS2 QDs/N, S@ GNA/cCNTs/GCE). The study demonstrated that the sensors were feasible, reproducible and selective for simultaneous detection of AZM and HCQ in various matrices [196].

### 5.1. Clinical Trial Status

Along with an increase in academic research efforts, only a few biopolymer-based aerogels have reached the clinical testing phase for drug delivery [197]. Additionally, aerogels have been suggested for potential clinical techniques, such as photothermal therapy (PTT). The epidermis acceptability of aerogel was evaluated using human volunteers recruited by PhD Trials^®^, Lda, a recognized clinical trial firm. After 48.5 h of exposure, assessments on the skin surface showed that none of the participants had a response, no matter what kind of aerogel was used. Alginate-aerogel derivatives from polysaccharide substances have been shown to improve mucus fluidity and sputum elimination in Level 2a clinical studies for the therapy of cystic fibrosis [198].

### 5.2. Challenging and Future Perspective

The understanding of self-healing materials technology has been aggressively explored to make the structure more robust and reliable. Encapsulating healing agents employing chemicals and advanced procedures has been demonstrated to be among of the most effective self-healing technologies. However, more effort must be made to apply self-healing technology to materials such as hydrogels and aerogels [199]. Currently, most of the aerogel study remains focused on preparation strategy and function analysis, including structure or property control. Then, much work continues to be carried out in the use of aerogel for many types of applications in wounds and tissue engineering as self-healing functions [145]. Self-repairing aerogel must overcome a variety of difficulties in the future to advance biomedical applications, including acceptable mechanical characteristics, biocompatibility, and clinical trial compliance. Furthermore, optimizing the biodegradability of self-healing aerogel used in biochemical engineering, artificial skin, and drug carrying is vital [200].

Additive manufacturing (AM) enables the production of custom-built products that would not be achievable using traditional fabrication methods. As a first phase, a digital 3D model was constructed utilizing either geometrical design from scratch or computer-aided an actual real object. Frequently, comprehensive knowledge of the underlying medical condition is necessary for the most successful treatment approach. Similarly, the utilization of synthetic 3D lung tissues using graphene-aerogel would allow a fundamental investigation for a realistic simulation of the necessary systems [201]. To solve a few of the constraints of the 3D printing technology, the optimization of cell-construct functional responses and the complex dynamics of natural tissue products should be investigated. Also, using 4D printing technology to create biopolymer-based scaffolds using aerogel materials with the advent of bioengineering technology is intriguing to discover. Therefore, biopolymers may become essential elements in future formulations of bio-ink, demonstrating their enormous promise in 4D bioprinting for bioengineering tissue and gene therapy [147].

Based on their ingredients, several types of aerogels are recognized as safe, nonetheless, on occasion, toxic occurrences have been reported. In some instances, the original aerogel has needed to be modified using more biocompatible elements to prevent toxicity. Consequently, non-invasive disease detection tests must be undertaken, not only to exclude any possible hazardous occurrence and describe the safe dosage but also to enhance the design of the aerogel and boost its biocompatibility [202].

Medical imaging is essential to public health because it provides early disease prediction and the most effective and secure treatment plan. A critical step in developing an implant-based biomaterial is the capability to photograph an implant on the outside of the body without injuring the patient. Aerogel acoustic absorption has been found as a potential method for non-invasive and speedy imaging of aerogel-based implants. This substance is appropriate for usage in the peripheral body area, specifically in the neurological system [48]. For example, it was necessary to investigate cellulose-based fluorescent materials by creating and exploiting fluorescent-based biomaterials for cell bioimaging [203,204].

Based on their classification and synthesis techniques, aerogels’ implications have been studied and explored. However, there are still a lot of challenges that must be resolved. The cost-effective and mass manufacture of high-quality aerogels remains a significant problem. To increase scale and lower cost, efforts have been made to optimize the synthesis method. As a result, ambient drying, modified super critical drying, and freeze drying (lyophilization) were employed. Unfortunately, it was difficult to entirely retain the gel’s microstructure under the drying conditions; thus, damage frequently happened [8]. Additionally, although there has been substantial advancement in the design and biological analysis of aerogels, additional work is still required to address the difficulties in the commercialization and clinical application of this complex class of materials. The development of the polymer- and non-polymer-based aerogels at the pre- and clinical phases will be crucial to their commercial success. Throughout this journey, reducing manufacturing expenses and enhancing the biological characteristics of aerogels will both be crucial [205]. Furthermore, despite the fact that aerogel is well recognised and is gaining more attention, aerogels’ potential in the medical field has not been fully investigated. The use of aerogels in this application, for instance, is not fully utilised even though it is known from their properties that they are essential in regenerative medicine. To produce biomaterials that can be commercialized, there is still a lot of works to be accomplished and a variety of courses to pursue. Despite the recent development of novel aerogel types, new species of single-component or hybrid aerogels still need to be developed [206].

## 6. Conclusions

Aerogel has outstanding features such as large surface area, light weight, high porosity, low density, and superior thermal insulation, making it suitable for a wide range of applications. Aerogels can be used in biomedicine due to their chemical, physical, and physicochemical properties that can be easily modified. This review highlights the performance of aerogels towards drug delivery, toxicity, wound healing, antioxidant, biocompatibility, and status reports in the case of COVID-19. As a result, aerogels are superior to other nanostructured materials due to their remarkable characteristics, which make them useful for drug delivery vehicles and tissue scaffolding. However, as far as we are concerned, the clinical status of aerogel is still far from satisfactory. Aerogels are gaining popularity as a replacement multifunctional biomaterial for promoting human health. Recently, advanced research in more depth in fields such as self-healing, additive manufacturing (AM) technology, toxicity, and fluorescent-based aerogels needs to be further explored.

## Figures and Tables

**Table 1:** Different types of aerogels with their respective properties.

Types	Main Component	Properties	Weakness	Methods for Improvement	Applications	References
Silica	Tetraethylorthosilicate (TEOS) and methyltrimethoxysilane (MTMS)	Low heat conductivity, large built-up area, low density	Fragile, have poor mechanical properties and require a lengthy processing technique	Use precursors in the backbone, surface-crosslinking with a polymer, prolonged aging incorporating, polymerizing	Photocatalysts, Thermal insulation, absorbent pollutants	[15,43]
Polymer	Cellulose/ conducting polymer	High moduli and fatigue resistance	Monolithic, prone to defects, length processing and costly	Usage of synthetic polymer	Additives (foods, cosmetics) construction, materials, drug delivery carrier	[30,44]
Carbon	Carbon/CNT/ graphene	High specific surface area and porosity, low density, good electrical conductor, good chemical stability, and hydrophobicity,	Low electrical conductivities and reduced heat transmission via the aerogel backbone phase with related organic precursor	Focused on carbon aerogel-based biomass	Electrodes, in supercapacitors, adsorbents for phenol	[39,45]
Inorganic	Oxide/ metallic/ chalcogenide	Ultra-high surface area and high open porosity	High production cost	Hybrid aerogel formation	Energy conversion, storage application	[46,47]
Organic	Biopolymer	High compressive strength, high surface area	Poor mechanical properties	Incorporated with inorganic fillers	Biosensor, Medical implantable device.	[48]

**Table 3:** Previous studies on wound healing and antimicrobial activity for various types of aerogels.

	Type of Aerogels	Material	Enhancement Technique	Remarks	Reference	Year
1	Polymer	Chitosan	Sol–gel method	Efficient in reducing bacterial loads at the wound site	[97]	2018
2	Polymer	Chitosan	Microwave-assisted conditions using biocompatible crosslinking agent	Had superior antibacterial properties against mentioned bacteria	[84]	2019
3	Inorganic	Silica	Surface modification in the gas phase	The chlorhexidine-loaded aerogel confirmed its potency in the elimination of *E. coli*	[64]	2019
4	Carbon	Graphene	Hydrothermal and postpyrolysis process	Complete wound-healing efficiency within 12 h	[111]	2019
5	Polymer	Starch	Physical crosslinking via freeze–thaw technique	Exhibited excellently antimicrobial activity against mentioned bacterial	[112]	2019
6	Polymer	Alginate	Supercritical impregnation of mesoglycan (MSG)	Supercritical impregnation is suitable to obtain MSG-loaded systems	[88]	2020
7	Polymer	Alginate, chitosan	Emulsion gelation	Percentages of recovered scratch area higher than the untreated control	[85]	2020
8	Carbon	Graphene	Carbonization	Exhibited excellent performance for the simultaneous elimination of *S. aureus.*	[70]	2019
9	Polymer	Chitosan	Electrophoretic deposition at low voltage	Accelerate wound healing and reduce the scar area	[92]	2019
10	Polymer	Cellulose	Freeze drying	High antibiotic activity against *S. aureus*	[104]	2020
11	Polymer	Alginate, chitosan	Sol–gel method followed by freeze-drying process	Stronger antibacterial activities against *S. aureus* and *E. coli*	[108]	2021
12	Polysaccharide	Hyaluronic acid (HA)	Electrospray method	HA aerogel bind and kill mycobacteria	[99]	2021
13	Polymer	Methoxy polyethylene glycol-polycaprol actone	Electrospinning, homogeneous dispersion, freeze-drying, and heat treatment	Good antimicrobial activity	[109]	2021
14	Polymer	Nanocellulose	Freeze-drying	Excellent and long-term antimicrobial activity against both *S. aureus* (gram-positive) and *E. coli* (gram-negative)	[113]	2021
15	Polymer	CNF, Chitosan	High pressure homogenization and freeze- drying	Bacterial reduction test *E. coli* and *S. aureus*	[110]	2021
16	Polymer	Alginate, chitosan	Sol–gel and supercritical fluid	Long time and safety to the wound surface	[98]	2021
17	Polymer	Chitosan	Freeze-drying	Has excellent antibacterial to promote woung healing	[102]	2021
18	Inorganic	Silica	Crystallization from supercritical solutions	95% inhibition rate even after ∼90% of cinnamaldehyde (CA) as antibacterial agent is released.	[96]	2022
19	Polymer	Chitosan	Casting method	Highly effective towards *E. coli* and *S. aureus* as antibacterial agents	[105]	2020
20	Polymer	Alginate	Maillard reaction and freeze-drying	Excellent antimicrobial activities against *S. aureus* and *E. coli*	[114]	2020
21	Polysaccharide	Polyvinyl alcohol (PVA)	Freeze drying/cross-linking process	Exhibited good antibacterial capability	[103]	2022
22	Polymer	Chitosan,	Addition and lyophilization	Good antimicrobial properties	[106]	2022

**Table 4:** Studies on anti-toxicities of aerogels.

	Types	Materials	Advanced Method	Remarks	Reference	Year
1	Polymer	Chitosan	Addition	High antioxidant effect	[124]	2018
2	Polymer	Cellulose	Valorization	High antioxidant capacity	[125]	2019
3	Biopolymer	Alginate, pectin	Crosslinking with divalent cation (Ca^2+^), sol–gel, freeze- drying process	Stronger antioxidant activity	[126]	2019
4	Polymer	Cellulose	Ultra-turrax homogenization	High antioxidant capacity	[127]	2019
5	Composite	Chitosan, okra powder, nano- silicon	Casting	Scavenging rate of about 2.05%	[105]	2020
6	Polymer	Cellulose	Valorization	High antioxidant activity	[128]	2020
7	Polymer	Chitosan, collagen	Solvent casting technique	Antioxidants properties show usefulness for pharmaceutical and cosmetic research	[129]	2020
8	Polymer	Phenolic	Emulsion-gelation	No antioxidant capacity	[130]	2020
9	Polymer	Cellulose	Ultra-turrax homogenization	Positive inhibition effect	[131]	2021
10	Polymer	Cellulose, alginate	Freeze-drying	Better antioxidant activity without Ca^2+^ crosslinking	[132]	2021
11	Polymer	Corn starch	Gelatinization	Presented great antioxidant activity	[133]	2021
12	Polymer	Chitosan	Freeze drying	Has excellent antioxidant activity	[102]	2021
13	Polymer	Cellulose, chitosan, alginate	Co-grinding	High antioxidant capacity	[134]	2021	
14	Polymer	Silk fibroin	Desalting, gelation, freeze- drying	Maintained the antioxidant activity of polysaccharide	[135]	2021	
15	Polysaccharide	Citrus, pectin, cellulose nanofibre	Pickering emulsion template	Antioxidant capacity of were maintained	[136]	2022	
16	Polymer	Pectin, lentil protein, flower oil	Pre-homogenization, mechanical stirring	Ultrasonic treatment decreased the antioxidant activity	[137]	2022	

**Table 5:** Previous works related to antioxidant activity for various types of aerogels.

	Types	Materials	Advanced Method	Remarks	Reference	Year
1	Polymer	Chitosan	Addition	Significant increased antioxidant	[124]	2018
2	Polymer	Cellulose	Valorization	High antioxidant capacity	[125]	2019
3	Biopolymer	Alginate, pectin	Crosslinking with divalent cation (Ca^2+^), sol–gel, freeze- drying process	Stronger antioxidant activity	[126]	2019
4	Polymer	Cellulose	Ultra-turrax homogenization	High antioxidant capacity	[127]	2019
5	Composite	Chitosan	Casting	Scavenging rate of 2.05%	[105]	2020
6	Polymer	Cellulose	Valorization	High antioxidant activity	[128]	2020
7	Polymer	Chitosan, collagen	Solvent casting	Antioxidants properties show usefulness for pharmaceutical and cosmetic research	[129]	2020
8	Polymer	Pectin	Emulsion-gelation	Positive antioxidant capacity	[130]	2020
9	Polymer	Cellulose	Ultra-turrax homogenization	Positive inhibition effect	[131]	2021
10	Polymer	Cellulose, alginate	Freeze-drying	Better antioxidant activity without Ca^2+^ crosslinking	[132]	2021
11	Polymer	Corn starch	Gelatinization	Presented great antioxidant activity	[133]	2021
12	Polymer	Chitosan	Freeze drying	Has excellent antioxidant activity	[102]	2021
13	Polymer	Cellulose, chitosan, alginate	Co-grinding	High antioxidant capacity	[134]	2021
14	Polymer	Silk fibroin	Desalting, gelation, freeze drying	Maintained the antioxidant activity of polysaccharide	[135]	2021
15	Polymer	Pectin, lentil protein, flower oil	Pre-homogenization, mechanical stirring	Antioxidant activity increased	[137]	2022

## Data Availability

The data is unavailable.

## References

[1] Lin J., Li G., Liu W., Qiu R., Wei H., Zong K., Cai X. (2021). A review of recent progress on the silica aerogel monoliths: Synthesis, reinforcement, and applications. J. Mater. Sci..

[2] Yang J., Li Y., Zheng Y., Xu Y., Zheng Z., Chen X., Liu W. (2019). Versatile aerogels for sensors. Small.

[3] Muhammad A., Lee D., Shin Y., Park J. (2021). Recent progress in polysaccharide aerogels: Their synthesis, application, and future outlook. Polymers.

[4] Soorbaghi F.P., Isanejad M., Salatin S., Ghorbani M., Jafari S., Derakhshankhah H. (2019). Bioaerogels: Synthesis approaches, cellular uptake, and the biomedical applications. Biomed. Pharmacother..

[5] Azum N., Rub M.A., Khan A., Khan A.A.P., Asiri A.M. (2021). Aerogel applications and future aspects. Advances in Aerogel Composites for Environmental Remediation.

[6] Ramesh M., Rajeshkumar L., Balaji D. (2021). Aerogels for insulation applications. Mater. Res. Found.

[7] Long L.-Y., Weng Y.-X., Wang Y.-Z. (2018). Cellulose aerogels: Synthesis, applications, and prospects. Polymers.

[8] Noman M.T., Amor N., Ali A., Petrik S., Coufal R., Adach K., Fijalkowski M. (2021). Aerogels for Biomedical, Energy and Sensing Applications. Gels.

[9] Karamikamkar S., Naguib H.E., Park C.B. (2020). Advances in precursor system for silica-based aerogel production toward improved mechanical properties, customized morphology, and multifunctionality: A review. Adv. Colloid Interface Sci..

[10] Liu Z., Ran Y., Xi J., Wang J. (2020). Polymeric hybrid aerogels and their biomedical applications. Soft Matter.

[11] Inshakova E., Inshakova A., Goncharov A. (2020). Engineered nanomaterials for energy sector: Market trends, modern applications and future prospects. IOP Conf. Ser. Mater. Sci. Eng..

[12] García-González C.A., Budtova T., Durães L., Erkey C., Del Gaudio P., Gurikov P., Koebel M., Liebner F., Neagu M., Smirnova I. (2019). An opinion paper on aerogels for biomedical and environmental applications. Molecules.

[13] Li Z., Zhao S., Koebel M.M., Malfait W.J. (2020). Silica aerogels with tailored chemical functionality. Mater. Des..

[14] Montes S., Maleki H. (2020). Aerogels and their applications. Colloidal Metal Oxide Nanoparticles.

[15] Zhao S., Siqueira G., Drdova S., Norris D., Ubert C., Bonnin A., Galmarini S., Ganobjak M., Pan Z., Brunner S. (2020). Additive manufacturing of silica aerogels. Nature.

[16] Saoud K.M., Saeed S., Bertino M.F., White L.S. (2018). Fabrication of strong and ultra-lightweight silica-based aerogel materials with tailored properties. J. Porous Mater..

[17] Wang Q., Yu H., Zhang Z., Zhao Y., Wang H. (2020). One-pot synthesis of polymer-reinforced silica aerogels from high internal phase emulsion templates. J. Colloid Interface Sci..

[18] Posada L.F., Carroll M.K., Anderson A.M., Bruno B.A. (2019). Inclusion of Ceria in Alumina- and Silica-Based Aerogels for Catalytic Applications. J. Supercrit. Fluids.

[19] Rezaei S., Zolali A.M., Jalali A., Park C.B. (2020). Novel and simple design of nanostructured, super-insulative and flexible hybrid silica aerogel with a new macromolecular polyether-based precursor. J. Colloid Interface Sci..

[20] Karamikamkar S., Abidli A., Behzadfar E., Rezaei S., Naguib H.E., Park C.B. (2019). The effect of graphene-nanoplatelets on gelation and structural integrity of a polyvinyltrimethoxysilane-based aerogel. RSC Adv..

[21] Choi H., Parale V.G., Kim T., Choi Y.-S., Tae J., Park H.-H. (2020). Structural and mechanical properties of hybrid silica aerogel formed using triethoxy (1-phenylethenyl) silane. Microporous Mesoporous Mater..

[22] Li Y., Liu X., Nie X., Yang W., Wang Y., Yu R., Shui J. (2019). Multifunctional organic–inorganic hybrid aerogel for self-cleaning, heat-insulating, and highly efficient microwave absorbing material. Adv. Funct. Mater..

[23] Tiryaki E., Elalmis Y.B., Ikizler B.K., Yücel S. (2020). Novel organic/inorganic hybrid nanoparticles as enzyme-triggered drug delivery systems: Dextran and Dextran aldehyde coated silica aerogels. J. Drug Deliv. Sci. Technol..

[24] Tian J., Yang Y., Xue T., Chao G., Fan W., Liu T. (2022). Highly flexible and compressible polyimide/silica aerogels with integrated double network for thermal insulation and fire-retardancy. J. Mater. Sci. Technol..

[25] Bonab S.A., Moghaddas J., Rezaei M. (2019). In-situ synthesis of silica aerogel/polyurethane inorganic-organic hybrid nanocomposite foams: Characterization, cell microstructure and mechanical properties. Polymer.

[26] Karamikamkar S., Fashandi M., Naguib H.E., Park C.B. (2020). In Situ Interface Design in Graphene-Embedded Polymeric Silica Aerogel with Organic/Inorganic Hybridization. ACS Appl. Mater. Interfaces.

[27] Zhang Y.-G., Zhu Y.-J., Xiong Z.-C., Wu J., Chen F. (2018). Bioinspired ultralight inorganic aerogel for highly efficient air filtration and oil–water separation. ACS Appl. Mater. Interfaces.

[28] Cho H.-J., Kim I.-D., Jung S.M. (2020). Multifunctional Inorganic Nanomaterial Aerogel Assembled into fSWNT Hydrogel Platform for Ultraselective NO2 Sensing. ACS Appl. Mater. Interfaces.

[29] Liu Q., Yan K., Chen J., Xia M., Li M., Liu K., Wang D., Wu C., Xie Y. (2021). Recent advances in novel aerogels through the hybrid aggregation of inorganic nanomaterials and polymeric fibers for thermal insulation. Aggregate.

[30] Arabkhani P., Asfaram A. (2020). Development of a novel three-dimensional magnetic polymer aerogel as an efficient adsorbent for malachite green removal. J. Hazard. Mater..

[31] Heidarshenas M., Kokabi M., Hosseini H. (2019). Shape memory conductive electrospun PVA/MWCNT nanocomposite aerogels. Polym. J..

[32] Pantoja M., Boynton N., Cavicchi K.A., Dosa B., Cashman J.L., Meador M.A.B. (2019). Increased flexibility in polyimide aerogels using aliphatic spacers in the polymer backbone. ACS Appl. Mater. Interfaces.

[33] Zuo L., Zhang Y., Zhang L., Miao Y.-E., Fan W., Liu T. (2015). Polymer/Carbon-Based Hybrid Aerogels: Preparation, Properties and Applications. Materials.

[34] Zhang X., Li W., Song P., You B., Sun G. (2020). Double-cross-linking strategy for preparing flexible, robust, and multifunctional polyimide aerogel. Chem. Eng. J..

[35] Zu G., Kanamori K., Maeno A., Kaji H., Nakanishi K. (2018). Superflexible Multifunctional Polyvinylpolydimethylsiloxane-Based Aerogels as Efficient Absorbents, Thermal Superinsulators, and Strain Sensors. Angew. Chem. Int. Ed..

[36] Liu Z., Zhang S., He B., Wang S., Kong F. (2021). Synthesis of cellulose aerogels as promising carriers for drug delivery: A review. Cellulose.

[37] Arenillas A., Menéndez J.A., Reichenauer G., Celzard A., Fierro V., Hodar F., Bailón E., Job N. (2019). Properties of Carbon Aerogels and Their Organic Precursors. Organic and Carbon Gels.

[38] Xu X., Li J., Li Y., Ni B., Liu X., Pan L., Ahualli S., Delgado Á.V. (2018). Chapter 4—Selection of Carbon Electrode Materials. Interface Science and Technology.

[39] Lee J.-H., Park S.-J. (2020). Recent advances in preparations and applications of carbon aerogels: A review. Carbon.

[40] Gong C., Ni J.-P., Tian C., Su Z.-H. (2021). Research in porous structure of cellulose aerogel made from cellulose nanofibrils. Int. J. Biol. Macromol..

[41] Lai K.C., Hiew B.Y.Z., Lee L.Y., Gan S., Thangalazhy-Gopakumar S., Chiu W.S., Khiew P.S. (2019). Ice-templated graphene oxide/chitosan aerogel as an effective adsorbent for sequestration of metanil yellow dye. Bioresour. Technol..

[42] Berglund L., Nissilä T., Sivaraman D., Komulainen S., Telkki V.-V., Oksman K. (2021). Seaweed-Derived Alginate–Cellulose Nanofiber Aerogel for Insulation Applications. ACS Appl. Mater. Interfaces.

[43] Chen Y., Hendrix Y., Schollbach K., Brouwers H. (2020). A silica aerogel synthesized from olivine and its application as a photocatalytic support. Constr. Build. Mater..

[44] Paraskevopoulou P., Chriti D., Raptopoulos G., Anyfantis G.C. (2019). Synthetic polymer aerogels in particulate form. Materials.

[45] Sam D.K., Sam E.K., Durairaj A., Lv X., Zhou Z., Liu J. (2020). Synthesis of biomass-based carbon aerogels in energy and sustainability. Carbohydr. Res..

[46] Alwin S., Sahaya Shajan X. (2020). Aerogels: Promising nanostructured materials for energy conversion and storage applications. Mater. Renew. Sustain. Energy.

[47] Korkmaz S., Kariper İ.A. (2020). Graphene and graphene oxide based aerogels: Synthesis, characteristics and supercapacitor applications. J. Energy Storage.

[48] El-Naggar M.E., Othman S.I., Allam A.A., Morsy O.M. (2020). Synthesis, drying process and medical application of polysaccharide-based aerogels. Int. J. Biol. Macromol..

[49] Babiarczuk B., Lewandowski D., Szczurek A., Kierzek K., Meffert M., Gerthsen D., Kaleta J., Krzak J. (2020). Novel approach of silica-PVA hybrid aerogel synthesis by simultaneous sol-gel process and phase separation. J. Supercrit. Fluids.

[50] Barrios E., Fox D., Li Sip Y.Y., Catarata R., Calderon J.E., Azim N., Afrin S., Zhang Z.Y., Zhai L. (2019). Nanomaterials in advanced, high-performance aerogel composites: A review. Polymers.

[51] Mekonnen B.T., Ding W., Liu H., Guo S., Pang X., Ding Z., Seid M.H. (2021). Preparation of aerogel and its application progress in coatings: A mini overview. J. Leather Sci. Eng..

[52] Dervin S., Pillai S.C. (2017). An introduction to sol-gel processing for aerogels. Sol-Gel Materials for Energy, Environment and Electronic Applications.

[53] Shi W., Ching Y.C., Chuah C.H. (2021). Preparation of aerogel beads and microspheres based on chitosan and cellulose for drug delivery: A review. Int. J. Biol. Macromol..

[54] Zhao C., Li Y., Ye W., Shen X., Yuan X., Ma C., Cao Y. (2021). Performance regulation of silica aerogel powder synthesized by a two-step Sol-gel process with a fast ambient pressure drying route. J. Non-Cryst. Solids.

[55] Zhai S., Yu K., Meng C., Wang H., Fu J. (2022). Eco-friendly approach for preparation of hybrid silica aerogel via freeze drying method. J. Mater. Sci..

[56] Berardi U., Zaidi S.M. (2019). Characterization of commercial aerogel-enhanced blankets obtained with supercritical drying and of a new ambient pressure drying blanket. Energy Build..

[57] Huang Y., Zhou T., He S., Xiao H., Dai H., Yuan B., Chen X., Yang X. (2019). Flame-retardant polyvinyl alcohol/cellulose nanofibers hybrid carbon aerogel by freeze drying with ultra-low phosphorus. Appl. Surf. Sci..

[58] Mißfeldt F., Gurikov P., Lölsberg W., Weinrich D., Lied F., Fricke M., Smirnova I. (2020). Continuous supercritical drying of aerogel particles: Proof of concept. Ind. Eng. Chem. Res..

[59] Çok S.S., Gizli N. (2020). Hydrophobic silica aerogels synthesized in ambient conditions by preserving the pore structure via two-step silylation. Ceram. Int..

[60] Long S., Wang H., He K., Zhou C., Zeng G., Lu Y., Cheng M., Song B., Yang Y., Wang Z. (2020). 3D graphene aerogel based photocatalysts: Synthesized, properties, and applications. Colloids Surf. A Physicochem. Eng. Asp..

[61] Jiao Y., Wan C., Bao W., Gao H., Liang D., Li J. (2018). Facile hydrothermal synthesis of Fe_3_O_4_ cellulose aerogel nanocomposite and its application in Fenton-like degradation of Rhodamine B. Carbohydr. Polym..

[62] Gupta P., Singh B., Agrawal A.K., Maji P.K. (2018). Low density and high strength nanofibrillated cellulose aerogel for thermal insulation application. Mater. Des..

[63] Zhu W., Jiang X., Liu F., You F., Yao C. (2020). Preparation of chitosan—Graphene oxide composite aerogel by hydrothermal method and its adsorption property of methyl orange. Polymers.

[64] Ganonyan N., Bar G., Gvishi R., Avnir D. (2021). Gradual hydrophobization of silica aerogel for controlled drug release. RSC Adv..

[65] Darmawan A., Rasyid S.A., Astuti Y. (2021). Modification of the glass surface with hydrophobic silica thin layers using tetraethylorthosilicate (TEOS) and trimethylchlorosilane (TMCS) precursors. Surf. Interface Anal..

[66] Shafi S., Zhao Y. (2020). Superhydrophobic, enhanced strength and thermal insulation silica aerogel/glass fiber felt based on methyltrimethoxysilane precursor and silica gel impregnation. J. Porous Mater..

[67] Zhang S., Xiao Q., Xiao Y., Li Z., Xiong S., Ding F., He J. (2022). Chitosan based aerogels with low shrinkage by chemical cross-linking and supramolecular interaction. Gels.

[68] Pinelli F., Nespoli T., Rossi F. (2021). Graphene oxide-chitosan aerogels: Synthesis, characterization, and use as adsorbent material for water contaminants. Gels.

[69] Gong Y., Yu Y., Kang H., Chen X., Liu H., Zhang Y., Sun Y., Song H. (2019). Synthesis and characterization of graphene oxide/chitosan composite aerogels with high mechanical performance. Polymers.

[70] Bajpai V.K., Shukla S., Khan I., Kang S.-M., Haldorai Y., Tripathi K.M., Jung S., Chen L., Kim T., Huh Y.S. (2019). A sustainable graphene aerogel capable of the adsorptive elimination of biogenic amines and bacteria from soy sauce and highly efficient cell proliferation. ACS Appl. Mater. Interfaces.

[71] Liu S., Zhou C., Mou S., Li J., Zhou M., Zeng Y., Luo C., Sun J., Wang Z., Xu W. (2019). Biocompatible graphene oxide–collagen composite aerogel for enhanced stiffness and in situ bone regeneration. Mater. Sci. Eng. C.

[72] Zhao T., Qiu Z., Zhang Y., Hu F., Zheng J., Lin C. (2021). Using a three-dimensional hydroxyapatite/graphene aerogel as a high-performance anode in microbial fuel cells. J. Environ. Chem. Eng..

[73] Parte F.G.B., Santoso S.P., Chou C.-C., Verma V., Wang H.-T., Ismadji S., Cheng K.-C. (2020). Current progress on the production, modification, and applications of bacterial cellulose. Crit. Rev. Biotechnol..

[74] Salehi M.H., Golbaten-Mofrad H., Jafari S.H., Goodarzi V., Entezari M., Hashemi M., Zamanlui S. (2021). Electrically conductive biocompatible composite aerogel based on nanofibrillated template of bacterial cellulose/polyaniline/nano-clay. Int. J. Biol. Macromol..

[75] Liu X., Zheng H., Li Y., Wang L., Wang C. (2019). A novel bacterial cellulose aerogel modified with PGMA via ARGET ATRP method for catalase immobilization. Fibers Polym..

[76] Reséndiz-Hernández P., Cortés-Hernández D., Méndez Nonell J., Escobedo-Bocardo J. (2014). Bioactive and biocompatible silica/pseudowollastonite aerogels. Adv. Sci. Technol..

[77] Lázár I., Forgács A., Horváth A., Király G., Nagy G., Len A., Dudás Z., Papp V., Balogh Z., Moldován K. (2020). Mechanism of hydration of biocompatible silica-casein aerogels probed by NMR and SANS reveal backbone rigidity. Appl. Surf. Sci..

[78] Sani N.S., Malek N.A.N.N., Jemon K., Kadir M.R.A., Hamdan H. (2020). In vitro bioactivity and osteoblast cell viability studies of hydroxyapatite-incorporated silica aerogel. J. Sol-Gel Sci. Technol..

[79] Qin L., He Y., Zhao X., Zhang T., Qin Y., Du A. (2020). Preparation, characterization, and in vitro sustained release profile of resveratrol-loaded silica aerogel. Molecules.

[80] Follmann H.D., Oliveira O.N., Martins A.C., Lazarin-Bidóia D., Nakamura C.V., Rubira A.F., Silva R., Asefa T. (2020). Nanofibrous silica microparticles/polymer hybrid aerogels for sustained delivery of poorly water-soluble camptothecin. J. Colloid Interface Sci..

[81] Király G., Egu J.C., Hargitai Z., Kovács I., Fábián I., Kalmár J., Szemán-Nagy G. (2021). Mesoporous Aerogel Microparticles Injected into the Abdominal Cavity of Mice Accumulate in Parathymic Lymph Nodes. Int. J. Mol. Sci..

[82] Wang X., Wang J., Feng S., Zhang Z., Wu C., Zhang X., Kang F. (2019). Nano-porous silica aerogels as promising biomaterials for oral drug delivery of paclitaxel. J. Biomed. Nanotechnol..

[83] Egu J., Moldován K., Herman P., István F., Kalmár J., Fenyvesi F. (2022). 6ER-017 Prevention of extravasation by the local application of hybrid aerogel microparticles as drug delivery systems for cervical cancer chemotherapy. BMJ.

[84] Piątkowski M., Radwan-Pragłowska J., Janus Ł., Bogdał D., Matysek D., Cablik V. (2019). Microwave-assisted synthesis and characterization of chitosan aerogels doped with Au-NPs for skin regeneration. Polym. Test..

[85] Batista M., Gonçalves V., Gaspar F., Nogueira I., Matias A., Gurikov P. (2020). Novel alginate-chitosan aerogel fibres for potential wound healing applications. Int. J. Biol. Macromol..

[86] Alnaief M., Obaidat R.M., Alsmadi M.t.M. (2020). Preparation of hybrid alginate-chitosan aerogel as potential carriers for pulmonary drug delivery. Polymers.

[87] Zhang Y., Liu Y., Guo Z., Li F., Zhang H., Bai F., Wang L. (2020). Chitosan-based bifunctional composite aerogel combining absorption and phototherapy for bacteria elimination. Carbohydr. Polym..

[88] Franco P., Pessolano E., Belvedere R., Petrella A., De Marco I. (2020). Supercritical impregnation of mesoglycan into calcium alginate aerogel for wound healing. J. Supercrit. Fluids.

[89] Athamneh T., Amin A., Benke E., Ambrus R., Leopold C.S., Gurikov P., Smirnova I. (2019). Alginate and hybrid alginate-hyaluronic acid aerogel microspheres as potential carrier for pulmonary drug delivery. J. Supercrit. Fluids.

[90] Mahmoudpour M., Dolatabadi J.E.-N., Hasanzadeh M., Soleymani J. (2021). Carbon-based aerogels for biomedical sensing: Advances toward designing the ideal sensor. Adv. Colloid Interface Sci..

[91] Tevlek A., Atya A.M.N., Almemar M., Duman M., Gokcen D., Ganin A.Y., Yiu H.H.P., Aydin H.M. (2020). Synthesis of conductive carbon aerogels decorated with β-tricalcium phosphate nanocrystallites. Sci. Rep..

[92] Guo X., Xu D., Zhao Y., Gao H., Shi X., Cai J., Deng H., Chen Y., Du Y. (2019). Electroassembly of chitin nanoparticles to construct freestanding hydrogels and high porous aerogels for wound healing. ACS Appl. Mater. Interfaces.

[93] Song X., Huang X., Li Z., Li Z., Wu K., Jiao Y., Zhou C. (2019). Construction of blood compatible chitin/graphene oxide composite aerogel beads for the adsorption of bilirubin. Carbohydr. Polym..

[94] Rostamitabar M., Subrahmanyam R., Gurikov P., Seide G., Jockenhoevel S., Ghazanfari S. (2021). Cellulose aerogel micro fibers for drug delivery applications. Mater. Sci. Eng. C.

[95] Anastasova E.I., Belyaeva A.A., Tsymbal S.A., Vinnik D.A., Vinogradov V.V. (2022). Hierarchical Porous Magnetite Structures: From Nanoparticle Assembly to Monolithic Aerogels. J. Colloid Interface Sci..

[96] Xie H., He Z., Liu Y., Zhao C., Guo B., Zhu C., Xu J. (2022). Efficient antibacterial agent delivery by mesoporous silica aerogel. ACS Omega.

[97] López-Iglesias C., Barros J., Ardao I., Monteiro F.J., Alvarez-Lorenzo C., Gómez-Amoza J.L., García-González C.A. (2019). Vancomycin-loaded chitosan aerogel particles for chronic wound applications. Carbohydr. Polym..

[98] Gorshkova N., Brovko O., Palamarchuk I., Bogolitsyn K., Ivakhnov A. (2021). Preparation of bioactive aerogel material based on sodium alginate and chitosan for controlled release of levomycetin. Polym. Adv. Technol..

[99] Simonson A.W., Umstead T.M., Lawanprasert A., Klein B., Almarzooqi S., Halstead E.S., Medina S.H. (2021). Extracellular matrix-inspired inhalable aerogels for rapid clearance of pulmonary tuberculosis. Biomaterials.

[100] Yahya E.B., Jummaat F., Amirul A.A., Adnan A.S., Olaiya N.G., Abdullah C.K., Rizal S., Mohamad Haafiz M.K., Abdul Khalil H.P.S. (2020). A Review on Revolutionary Natural Biopolymer-Based Aerogels for Antibacterial Delivery. Antibiotics.

[101] Rashki S., Asgarpour K., Tarrahimofrad H., Hashemipour M., Ebrahimi M.S., Fathizadeh H., Khorshidi A., Khan H., Marzhoseyni Z., Salavati-Niasari M. (2021). Chitosan-based nanoparticles against bacterial infections. Carbohydr. Polym..

[102] Zhang K., Jiao X., Zhou L., Wang J., Wang C., Qin Y., Wen Y. (2021). Nanofibrous composite aerogel with multi-bioactive and fluid gating characteristics for promoting diabetic wound healing. Biomaterials.

[103] Yan Q., Long X., Zhang P., Lei W., Sun D., Ye X. (2022). Oxidized Bletilla rhizome polysaccharide-based aerogel with synergistic antibiosis and hemostasis for wound healing. Carbohydr. Polym..

[104] Revin V.V., Nazarova N.B., Tsareva E.E., Liyaskina E.V., Revin V.D., Pestov N.A. (2020). Production of bacterial cellulose aerogels with improved physico-mechanical properties and antibacterial effect. Front. Bioeng. Biotechnol..

[105] Lin D., Zheng Y., Huang Y., Ni L., Zhao J., Huang C., Chen X., Chen X., Wu Z., Wu D. (2020). Investigation of the structural, physical properties, antioxidant, and antimicrobial activity of chitosan-nano-silicon aerogel composite edible films incorporated with okara powder. Carbohydr. Polym..

[106] Chen L., Niu X., Fan X., Liu Y., Yang J., Xu X., Zhou G., Zhu B., Ullah N., Feng X. (2022). Highly absorbent antibacterial chitosan-based aerogels for shelf-life extension of fresh pork. Food Control.

[107] Lovskaya D., Menshutina N. (2020). Alginate-based aerogel particles as drug delivery systems: Investigation of the supercritical adsorption and in vitro evaluations. Materials.

[108] Pan J., Li Y., Chen K., Zhang Y., Zhang H. (2021). Enhanced physical and antimicrobial properties of alginate/chitosan composite aerogels based on electrostatic interactions and noncovalent crosslinking. Carbohydr. Polym..

[109] Jia J., Wang C. (2019). A facile restructuring of 3D high water absorption aerogels from methoxy polyethylene glycol-polycaprolactone (mPEG-PCL) nanofibers. Mater. Sci. Eng. C.

[110] Rizal S., Yahya E.B., Abdul Khalil H., Abdullah C., Marwan M., Ikramullah I., Muksin U. (2021). Preparation and characterization of nanocellulose/chitosan aerogel scaffolds using chemical-free approach. Gels.

[111] Shukla S., Khan I., Bajpai V.K., Lee H., Kim T., Upadhyay A., Huh Y.S., Han Y.-K., Tripathi K.M. (2019). Sustainable graphene aerogel as an ecofriendly cell growth promoter and highly efficient adsorbent for histamine from red wine. ACS Appl. Mater. Interfaces.

[112] Chin S.F., Romainor A.N.B., Pang S.C., Lihan S. (2019). Antimicrobial starch-citrate hydrogel for potential applications as drug delivery carriers. J. Drug Deliv. Sci. Technol..

[113] Saini A., Yadav C., Sethi S.K., Xue B.-L., Xia Y., Li K., Manik G., Li X. (2021). Microdesigned nanocellulose-based flexible antibacterial aerogel architectures impregnated with bioactive Cinnamomum cassia. ACS Appl. Mater. Interfaces.

[114] Chen K., Zhang H. (2020). Fabrication of oleogels via a facile method by oil absorption in the aerogel templates of protein–polysaccharide conjugates. ACS Appl. Mater. Interfaces.

[115] Ko E., Kim H. (2020). Preparation of chitosan aerogel crosslinked in chemical and ionical ways by non-acid condition for wound dressing. Int. J. Biol. Macromol..

[116] Figueroa T., Carmona S., Guajardo S., Borges J., Aguayo C., Fernández K. (2021). Synthesis and characterization of graphene oxide chitosan aerogels reinforced with flavan-3-ols as hemostatic agents. Colloids Surf. B Biointerfaces.

[117] Borges-Vilches J., Figueroa T., Guajardo S., Aguayo C., Fernández K. (2021). Improved hemocompatibility for gelatin-graphene oxide composite aerogels reinforced with proanthocyanidins for wound dressing applications. Colloids Surfaces B Biointerfaces.

[118] Wu Y., Jin M., Huang Y., Wang F. (2022). Insights into the Prospective Aerogel Scaffolds Composed of Chitosan/Aramid Nanofibers for Tissue Engineering. ACS Appl. Polym. Mater..

[119] Nagy G., Király G., Veres P., Lázár I., Fábián I., Bánfalvi G., Juhász I., Kalmár J. (2019). Controlled release of methotrexate from functionalized silica-gelatin aerogel microparticles applied against tumor cell growth. Int. J. Pharm..

[120] Alsmadi M.M., Obaidat R.M., Alnaief M., Albiss B.A., Hailat N. (2020). Development, in vitro characterization, and in vivo toxicity evaluation of chitosan-alginate nanoporous carriers loaded with cisplatin for lung cancer treatment. AAPS PharmSciTech.

[121] Wu X.-X., Zhang Y., Hu T., Li W.-X., Li Z.-L., Hu H.-J., Zhu S.-R., Chen W.-Z., Zhou C.-S., Jiang G.-B. (2021). Long-term antibacterial composite via alginate aerogel sustained release of antibiotics and Cu used for bone tissue bacteria infection. Int. J. Biol. Macromol..

[122] Liu Z., Zhang S., Gao C., Meng X., Wang S., Kong F. (2022). Temperature/pH-responsive carboxymethyl cellulose/poly (N-isopropyl acrylamide) interpenetrating polymer network aerogels for drug delivery systems. Polymers.

[123] Hu X., Wang Y., Zhang L., Xu M. (2021). Simple ultrasonic-assisted approach to prepare polysaccharide-based aerogel for cell research and histocompatibility study. Int. J. Biol. Macromol..

[124] Radwan-Pragłowska J., Piątkowski M., Janus Ł., Bogdał D., Matýsek D., Cablik V. (2018). Microwave-assisted synthesis and characterization of antioxidant chitosan-based aerogels for biomedical applications. Int. J. Polym. Anal. Charact..

[125] de Oliveira J.P., Bruni G.P., Fabra M.J., Zavareze E.D.R., López-Rubio A., Martínez-Sanz M. (2019). Development of food packaging bioactive aerogels through the valorization of Gelidium sesquipedale seaweed. Food Hydrocoll..

[126] Chen K., Zhang H. (2019). Alginate/pectin aerogel microspheres for controlled release of proanthocyanidins. Int. J. Biol. Macromol..

[127] Fontes-Candia C., Erboz E., Martínez-Abad A., López-Rubio A., Martínez-Sanz M. (2019). Superabsorbent food packaging bioactive cellulose-based aerogels from Arundo donax waste biomass. Food Hydrocoll..

[128] de Oliveira J.P., Bruni G.P., Fonseca L.M., da Silva F.T., da Rocha J.C., da Rosa Zavareze E. (2020). Characterization of aerogels as bioactive delivery vehicles produced through the valorization of yerba-mate (Illex paraguariensis). Food Hydrocoll..

[129] Thongchai K., Chuysinuan P., Thanyacharoen T., Techasakul S., Ummartyotin S. (2020). Characterization, release, and antioxidant activity of caffeic acid-loaded collagen and chitosan hydrogel composites. J. Mater. Res. Technol..

[130] Viganó J., Meirelles A.A.D., Náthia-Neves G., Baseggio A.M., Cunha R., Junior M.R.M., Meireles M.A.A., Gurikov P., Smirnova I., Martínez J. (2020). Impregnation of passion fruit bagasse extract in alginate aerogel microparticles. Int. J. Biol. Macromol..

[131] Benito-González I., López-Rubio A., Galarza-Jiménez P., Martínez-Sanz M. (2021). Multifunctional cellulosic aerogels from Posidonia oceanica waste biomass with antioxidant properties for meat preservation. Int. J. Biol. Macromol..

[132] Zhang A., Zou Y., Xi Y., Wang P., Zhang Y., Wu L., Zhang H. (2021). Fabrication and characterization of bamboo shoot cellulose/sodium alginate composite aerogels for sustained release of curcumin. Int. J. Biol. Macromol..

[133] Fonseca L.M., da Silva F.T., Bruni G.P., Borges C.D., da Rosa Zavareze E., Dias A.R.G. (2021). Aerogels based on corn starch as carriers for pinhão coat extract (Araucaria angustifolia) rich in phenolic compounds for active packaging. Int. J. Biol. Macromol..

[134] Coldebella R., Gentil M., Berger C., Costa H.D., Pedrazzi C., Labidi J., Delucis R., Missio A. (2021). Nanofibrillated Cellulose-Based Aerogels Functionalized with Tajuva (Maclura tinctoria) Heartwood Extract. Polymers.

[135] Zhu Y., Li J., Ma J., Lin Z., Lu X., Xiong Q., Qian Y., Yuan J., Ding S., Huang S. (2021). An effective, green and mild deproteinization method for polysaccharides of Ruditapes philippinarum by attapulgite-based silk fibroin composite aerogel. Int. J. Biol. Macromol..

[136] Wu W., Wu Y., Lin Y., Shao P. (2022). Facile fabrication of multifunctional citrus pectin aerogel fortified with cellulose nanofiber as controlled packaging of edible fungi. Food Chem..

[137] Mekala S., Silva E.K., Saldaña M.D. (2022). Ultrasound-assisted production of emulsion-filled pectin hydrogels to encapsulate vitamin complex: Impact of the addition of xylooligosaccharides, ascorbic acid and supercritical CO2 drying. Innov. Food Sci. Emerg. Technol..

[138] Găman A.M., Egbuna C., Găman M.-A. (2020). Natural bioactive lead compounds effective against haematological malignancies. Phytochemicals as Lead Compounds for New Drug Discovery.

[139] Penta S. (2015). Advances in Structure and Activity Relationship of Coumarin Derivatives.

[140] Noremylia M., Hassan M.Z., Ismail Z. (2022). Recent advancement in isolation, processing, characterization and applications of emerging nanocellulose: A review. Int. J. Biol. Macromol..

[141] García-González C.A., Sosnik A., Kalmár J., De Marco I., Erkey C., Concheiro A., Alvarez-Lorenzo C. (2021). Aerogels in drug delivery: From design to application. J. Control. Release.

[142] Berrio M., Oñate A., Salas A., Fernández K., Meléndrez M. (2021). Synthesis and applications of graphene oxide aerogels in bone tissue regeneration: A review. Mater. Today Chem..

[143] Witzler M., Büchner D., Shoushrah S.H., Babczyk P., Baranova J., Witzleben S., Tobiasch E., Schulze M. (2019). Polysaccharide-based systems for targeted stem cell differentiation and bone regeneration. Biomolecules.

[144] Kim S.-K., Murugan S.S., Dalavi P.A., Gupta S., Anil S., Seong G.H., Venkatesan J. (2022). Biomimetic chitosan with biocomposite nanomaterials for bone tissue repair and regeneration. Beilstein J. Nanotechnol..

[145] Zheng L., Zhang S., Ying Z., Liu J., Zhou Y., Chen F. (2020). Engineering of aerogel-based biomaterials for biomedical applications. Int. J. Nanomed..

[146] Huang G.-J., Yu H.-P., Wang X.-L., Ning B.-B., Gao J., Shi Y.-Q., Zhu Y.-J., Duan J.-L. (2021). Highly porous and elastic aerogel based on ultralong hydroxyapatite nanowires for high-performance bone regeneration and neovascularization. J. Mater. Chem. B.

[147] Yahya E.B., Amirul A.A., H.P.S. A.K., Olaiya N.G., Iqbal M.O., Jummaat F., A.K. A.S., Adnan A.S. (2021). Insights into the Role of Biopolymer Aerogel Scaffolds in Tissue Engineering and Regenerative Medicine. Polymers.

[148] Maleki H., Shahbazi M.-A., Montes S., Hosseini S.H., Eskandari M.R., Zaunschirm S., Verwanger T., Mathur S., Milow B., Krammer B. (2019). Mechanically Strong Silica-Silk Fibroin Bioaerogel: A Hybrid Scaffold with Ordered Honeycomb Micromorphology and Multiscale Porosity for Bone Regeneration. ACS Appl. Mater. Interfaces.

[149] Goimil L., Santos-Rosales V., Delgado A., Évora C., Reyes R., Lozano-Pérez A.A., Aznar-Cervantes S.D., Cenis J.L., Gómez-Amoza J.L., Concheiro A. (2019). scCO2-foamed silk fibroin aerogel/poly(ε-caprolactone) scaffolds containing dexamethasone for bone regeneration. J. CO2 Util..

[150] Osorio D.A., Lee B.E.J., Kwiecien J.M., Wang X., Shahid I., Hurley A.L., Cranston E.D., Grandfield K. (2019). Cross-linked cellulose nanocrystal aerogels as viable bone tissue scaffolds. Acta Biomater..

[151] Kamel R., Mabrouk M., El-Sayed S.A., Beherei H.H., Abouzeid R.E., El-Fadl M.T.A., Mahmoud A.A., Maged A. (2022). Nanofibrillated cellulose/glucosamine 3D aerogel implants loaded with rosuvastatin and bioactive ceramic for dental socket preservation. Int. J. Pharm..

[152] Elkhenany H., Bourdo S., Hecht S., Donnell R., Gerard D., Abdelwahed R., Lafont A., Alghazali K., Watanabe F., Biris A.S. (2017). Graphene nanoparticles as osteoinductive and osteoconductive platform for stem cell and bone regeneration. Nanomed. Nanotechnol. Biol. Med..

[153] Liu C., Wang S., Wang N., Yu J., Liu Y.-T., Ding B. (2022). From 1D Nanofibers to 3D Nanofibrous Aerogels: A Marvellous Evolution of Electrospun SiO2 Nanofibers for Emerging Applications. Nano-Micro Lett..

[154] Hegedűs C., Czibulya Z., Tóth F., Dezső B., Hegedűs V., Boda R., Horváth D., Csík A., Fábián I., Tóth-Győri E. (2022). The Effect of Heat Treatment of &beta;-Tricalcium Phosphate-Containing Silica-Based Bioactive Aerogels on the Cellular Metabolism and Proliferation of MG63 Cells. Biomedicines.

[155] Sathiyavimal S., Vasantharaj S., LewisOscar F., Selvaraj R., Brindhadevi K., Pugazhendhi A. (2020). Natural organic and inorganic–hydroxyapatite biopolymer composite for biomedical applications. Prog. Org. Coat..

[156] Zhu J., Xiong R., Zhao F., Peng T., Hu J., Xie L., Xie H., Wang K., Jiang C. (2020). Lightweight, High-Strength, and Anisotropic Structure Composite Aerogel Based on Hydroxyapatite Nanocrystal and Chitosan with Thermal Insulation and Flame Retardant Properties. ACS Sustain. Chem. Eng..

[157] Iglesias-Mejuto A., García-González C.A. (2022). 3D-Printed, Dual Crosslinked and Sterile Aerogel Scaffolds for Bone Tissue Engineering. Polymers.

[158] Chen Z.-J., Shi H.-H., Zheng L., Zhang H., Cha Y.-Y., Ruan H.-X., Zhang Y., Zhang X.-C. (2021). A new cancellous bone material of silk fibroin/cellulose dual network composite aerogel reinforced by nano-hydroxyapatite filler. Int. J. Biol. Macromol..

[159] Liu M., Shafiq M., Sun B., Wu J., Wang W., El-Newehy M., El-Hamshary H., Morsi Y., Ali O., Khan A. (2022). Composite Superelastic Aerogel Scaffolds Containing Flexible SiO 2 Nanofibers Promote Bone Regeneration. Adv. Healthc. Mater..

[160] Jodati H., Yilmaz B., Evis Z. (2021). In vitro and in vivo properties of graphene-incorporated scaffolds for bone defect repair. Ceram. Int..

[161] Qi X., Jiang F., Zhou M., Zhang W., Jiang X. (2021). Graphene oxide as a promising material in dentistry and tissue regeneration: A review. Smart Mater. Med..

[162] Shuai C., Yang F., Shuai Y., Peng S., Chen S., Deng Y., Feng P. (2022). Silicon dioxide nanoparticles decorated on graphene oxide nanosheets and their application in poly(l-lactic acid) scaffold. J. Adv. Res..

[163] Nie W., Peng C., Zhou X., Chen L., Wang W., Zhang Y., Ma P.X., He C. (2017). Three-dimensional porous scaffold by self-assembly of reduced graphene oxide and nano-hydroxyapatite composites for bone tissue engineering. Carbon.

[164] Asha S., Kumar G.V., Ananth A.N., Jose S., Rajan M.J. (2019). Investigations on Bio-mineralization of reduced graphene oxide aerogel in thepresence of various polymers. Mater. Today Proc..

[165] Asha S., Ananth A.N., Jose S.P., Rajan M.A.J. (2018). Reduced graphene oxide aerogel networks with soft interfacial template for applications in bone tissue regeneration. Appl. Nanosci..

[166] Bahrami S., Baheiraei N., Shahrezaee M. (2021). Biomimetic reduced graphene oxide coated collagen scaffold for in situ bone regeneration. Sci. Rep..

[167] Chang L.R., Marston G., Martin A. (2021). Anatomy, Cartilage.

[168] Salerno A., Pascual C.D. (2015). Bio-based polymers, supercritical fluids and tissue engineering. Process Biochem..

[169] Xu T., Ding Y., Liang Z., Sun H., Zheng F., Zhu Z., Zhao Y., Fong H. (2020). Three-dimensional monolithic porous structures assembled from fragmented electrospun nanofiber mats/membranes: Methods, properties, and applications. Prog. Mater. Sci..

[170] Huang J., Liu F., Su H., Xiong J., Yang L., Xia J., Liang Y. (2022). Advanced Nanocomposite Hydrogels for Cartilage Tissue Engineering. Gels.

[171] Bingül N.D., Öz Y.E., Şendemir A., Hameş E.E. (2022). Microbial biopolymers in articular cartilage tissue engineering. J. Polym. Res..

[172] Zhao X., Hu D.A., Wu D., He F., Wang H., Huang L., Shi D., Liu Q., Ni N., Pakvasa M. (2021). Applications of Biocompatible Scaffold Materials in Stem Cell-Based Cartilage Tissue Engineering. Front. Bioeng. Biotechnol..

[173] Chen Y., Shafiq M., Liu M., Morsi Y., Mo X. (2020). Advanced fabrication for electrospun three-dimensional nanofiber aerogels and scaffolds. Bioact. Mater..

[174] Dilamian M., Joghataei M., Ashrafi Z., Bohr C., Mathur S., Maleki H. (2021). From 1D electrospun nanofibers to advanced multifunctional fibrous 3D aerogels. Appl. Mater. Today.

[175] Chen W., Chen S., Morsi Y., El-Hamshary H., El-Newhy M., Fan C., Mo X. (2016). Superabsorbent 3D scaffold based on electrospun nanofibers for cartilage tissue engineering. ACS Appl. Mater. Interfaces.

[176] Yuan Z., Ren Y., Shafiq M., Chen Y., Tang H., Li B., EL-Newehy M., EL-Hamshary H., Morsi Y., Zheng H. (2022). Converging 3D printing and electrospinning: Effect of poly (L-lactide)/gelatin based short nanofibers aerogels on tracheal regeneration. Macromol. Biosci..

[177] Wang L., Zhang Y., Peng H., Chen R., Long Y., Liu Z., Yu T., Zhang Y. (2021). Electrospun gelatin polycaprolactone nanofiber aerogel combined with cartilage extracellular matrix for repair of cartilage injury in rabbits. Chin. J. Trauma.

[178] Zhang L., Fang J., Fu L., Chen L., Dai W., Huang H., Wang J., Zhang X., Cai Q., Yang X. (2021). Gradient fibrous aerogel conjugated with chemokine peptide for regulating cell differentiation and facilitating osteochondral regeneration. Chem. Eng. J..

[179] Hong J.-Y., Yun S., Wie J.J., Zhang X., Dresselhaus M.S., Kong J., Park H.S. (2016). Cartilage-inspired superelastic ultradurable graphene aerogels prepared by the selective gluing of intersheet joints. Nanoscale.

[180] Biswal T. (2021). Biopolymers for tissue engineering applications: A review. Mater. Today Proc..

[181] Lo S., Fauzi M.B. (2021). Current Update of Collagen Nanomaterials—Fabrication, Characterisation and Its Applications: A Review. Pharmaceutics.

[182] Qin D., Wang N., You X.-G., Zhang A.-D., Chen X.-G., Liu Y. (2022). Collagen-based biocomposites inspired by bone hierarchical structures for advanced bone regeneration: Ongoing research and perspectives. Biomater. Sci..

[183] Muñoz-Ruíz A., Escobar-García D.M., Quintana M., Pozos-Guillén A., Flores H. (2019). Synthesis and Characterization of a New Collagen-Alginate Aerogel for Tissue Engineering. J. Nanomater..

[184] Wei Z., Wu C., Li R., Yu D., Ding Q. (2021). Nanocellulose based hydrogel or aerogel scaffolds for tissue engineering. Cellulose.

[185] Mirtaghavi A., Luo J., Muthuraj R. (2020). Recent advances in porous 3d cellulose aerogels for tissue engineering applications: A review. J. Compos. Sci..

[186] Tang A., Ji J., Li J., Liu W., Wang J., Sun Q., Li Q. (2021). Nanocellulose/PEGDA Aerogels with Tunable Poisson’s Ratio Fabricated by Stereolithography for Mouse Bone Marrow Mesenchymal Stem Cell Culture. Nanomaterials.

[187] Li V.C.-F., Dunn C.K., Zhang Z., Deng Y., Qi H.J. (2017). Direct Ink Write (DIW) 3D Printed Cellulose Nanocrystal Aerogel Structures. Sci. Rep..

[188] Kuttor A., Szalóki M., Rente T., Kerényi F., Bakó J., Fábián I., Lázár I., Jenei A., Hegedüs C. (2014). Preparation and application of highly porous aerogel-based bioactive materials in dentistry. Front. Mater. Sci..

[189] Aminoroaya A., Bagheri R., Khorasani S.N., Talebi Z., Derakhshanfar P., Neisiany R.E. (2022). Mesoporous silica aerogel reinforced dental composite: Effects of microstructure and surface modification. J. Mech. Behav. Biomed. Mater..

[190] Cheng J., Deng Y., Tan Y., Li J., Fei Y., Wang C., Zhang J., Niu C., Fu Q., Lu L. (2022). Preparation of Silica Aerogel/Resin Composites and Their Application in Dental Restorative Materials. Molecules.

[191] Lázár I., Bereczki H.F., Manó S., Daróczi L., Deák G., Fábián I., Csernátony Z. (2015). Synthesis and study of new functionalized silica aerogel poly(methyl methacrylate) composites for biomedical use. Polym. Compos..

[192] Amiri P., Talebi Z., Semnani D., Bagheri R. (2019). Determination of the Optimized Conditions for Preparation of Bis-GMA Based Dental Composite Reinforced by a Nanostructure of Silica Aerogel. Nashrieh Shimi Va Mohandesi Shimi Iran.

[193] Lázár I., Kuttor A., Győri E., Veres P., Fábián I., Manó S., Hegedüs C. (2015). Preparation and characteristics of aerogel-based bioactive materials used in dentistry. Fogorv. Szle..

[194] Hegedűs V., Kerényi F., Boda R., Horváth D., Lázár I., Tóth-Győri E., Dezső B., Hegedus C. (2018). β-Tricalcium phosphate silica aerogel as an alternative bioactive ceramic for the potential use in dentistry. Adv. Appl. Ceram..

[195] Duong T., López-Iglesias C., Szewczyk P.K., Stachewicz U., Barros J., Alvarez-Lorenzo C., Alnaief M., García-González C.A. (2021). A pathway from porous particle technology toward tailoring aerogels for pulmonary drug administration. Front. Bioeng. Biotechnol..

[196] Mahnashi H.M., Mahmoud A.M., Alkahtani A.S., El-Wekil M.M. (2021). Simultaneous electrochemical detection of azithromycin and hydroxychloroquine based on VS2 QDs embedded N, S@ graphene aerogel/cCNTs 3D nanostructure. Microchem. J..

[197] Khalil H.A., Yahya E.B., Jummaat F., Adnan A., Olaiya N., Rizal S., Abdullah C., Pasquini D., Thomas S. (2023). Biopolymers based aerogels: A review on revolutionary solutions for smart therapeutics delivery. Prog. Mater. Sci..

[198] López-Iglesias C., Casielles A.M., Altay A., Bettini R., Alvarez-Lorenzo C., García-González C.A. (2019). From the printer to the lungs: Inkjet-printed aerogel particles for pulmonary delivery. Chem. Eng. J..

[199] Neisiany R.E., Khorasani S.N., Lee J.K.Y., Razavi J., Enayati M.S., Naeimirad M., Berto F., Ramakrishna S. (2021). Core-shell nanofibers for developing self-healing materials: Recent progress and future directions. Mater. Des. Process. Commun..

[200] Idumah C.I. (2021). Recent advancements in self-healing polymers, polymer blends, and nanocomposites. Polym. Polym. Compos..

[201] Bock S., Rades T., Rantanen J., Scherließ R. (2022). Additive Manufacturing in respiratory sciences-current applications and future prospects. Adv. Drug Deliv. Rev..

[202] Ferreira-Gonçalves T., Iglesias-Mejuto A., Linhares T., Coelho J.M.P., Vieira P., Faísca P., Catarino J., Pinto P., Ferreira D., Ferreira H.A. (2022). Biological Thermal Performance of Organic and Inorganic Aerogels as Patches for Photothermal Therapy. Gels.

[203] Nawaz H., Chen S., Li X., Zhang X., Zhang X., Wang J.-Q., Xu F. (2022). Cellulose-based environment-friendly smart materials for colorimetric and fluorescent detection of Cu2+/Fe3+ ions and their anti-counterfeiting applications. Chem. Eng. J..

[204] Zhang Z., Liu G., Li X., Zhang S., Lü X., Wang Y. (2020). Design and synthesis of fluorescent nanocelluloses for sensing and bioimaging applications. ChemPlusChem.

[205] Ferreira-Gonçalves T., Constantin C., Neagu M., Reis C.P., Sabri F., Simón-Vázquez R. (2021). Safety and efficacy assessment of aerogels for biomedical applications. Biomed. Pharmacother..

[206] Nita L.E., Ghilan A., Rusu A.G., Neamtu I., Chiriac A.P. (2020). New Trends in Bio-Based Aerogels. Pharmaceutics.

